# Descriptors for unprofessional behaviours of medical students: a systematic review and categorisation

**DOI:** 10.1186/s12909-017-0997-x

**Published:** 2017-09-15

**Authors:** Marianne Mak-van der Vossen, Walther van Mook, Stéphanie van der Burgt, Joyce Kors, Johannes C.F. Ket, Gerda Croiset, Rashmi Kusurkar

**Affiliations:** 1 Department of Research in Education, VUmc School of Medical Sciences, Amsterdam, the Netherlands; 2 LEARN! Research Institute for Education and Learning, VU University, Amsterdam, the Netherlands; 3 Department for General Practice and Elderly Care Management, VU Medical Center, Amsterdam, the Netherlands; 4 Department of Intensive Care Medicine, Maastricht University Medical Center, Maastricht, the Netherlands; 5 Department of Medical Education Development and Research, Faculty of Health, Medicine and Life Sciences, Maastricht University, Maastricht, the Netherlands; 6 AVAG Midwifery Academy Amsterdam Groningen, Amsterdam, the Netherlands; 7 Medical Library, University Library, Vrije Universiteit, Amsterdam, the Netherlands

**Keywords:** Medical education, Medical students, Humanities, Professionalism, Unprofessional behaviour, Professional misconduct, Systematic review

## Abstract

**Background:**

Developing professionalism is a core task in medical education. Unfortunately, it has remained difficult for educators to identify medical students’ *unprofessionalism*, because, among other reasons, there are no commonly adopted descriptors that can be used to document students’ unprofessional behaviour. This study aimed to generate an overview of descriptors for unprofessional behaviour based on research evidence of real-life unprofessional behaviours of medical students.

**Methods:**

A systematic review was conducted searching PubMed, Ebsco/ERIC, Ebsco/PsycINFO and Embase.com from inception to 2016. Articles were reviewed for admitted or witnessed unprofessional behaviours of undergraduate medical students.

**Results:**

The search yielded 11,963 different studies, 46 met all inclusion criteria. We found 205 different descriptions of unprofessional behaviours, which were coded into 30 different descriptors, and subsequently classified in four behavioural themes: *failure to engage*, *dishonest behaviour*, *disrespectful behaviour*, and *poor self-awareness*.

**Conclusions:**

This overview provides a common language to describe medical students’ unprofessional behaviour. The framework of descriptors is proposed as a tool for educators to denominate students’ unprofessional behaviours. The found behaviours can have various causes, which should be explored in a discussion with the student about personal, interpersonal and/or institutional circumstances in which the behaviour occurred. Explicitly denominating unprofessional behaviour serves two goals: [i] creating a culture in which unprofessional behaviour is acknowledged, [ii] targeting students who need extra guidance. Both are important to avoid unprofessional behaviour among future doctors.

**Electronic supplementary material:**

The online version of this article (10.1186/s12909-017-0997-x) contains supplementary material, which is available to authorized users.

## Background

Medical educators who observe professionalism lapses in their students do not always denominate these lapses directly and clearly in professionalism evaluations [1]. Evaluating professionalism is difficult, partly because educators are afraid to be subjective, but also because a commonly adopted language to describe *un*professionalism does not exist. Professionalism guidelines sometimes describe *normative unprofessional* behaviours, but these are not based on systematic empirical research on students’ *actual unprofessional* behaviours, as witnessed by medical educators, physicians, other health personnel, patients and students [2]. Should educators learn which behaviours are seen as unprofessional by peer educators and by students themselves, it might be easier for them to recognise and denominate unprofessional behaviours, and they might feel supported in acknowledging them [3].

Medical education must lay the foundation for the professional development of students through teaching and evaluating professionalism [4, 5]. Teaching professionalism is complex, as it requires strategies that explicitly as well as implicitly develop a learner’s knowledge, attitudes, judgment and skills [6]. Explicit teaching of professionalism includes the decisive actions taken by the medical school, while implicit teaching includes supervisors’ tacit modeling. This tacit modelling, the hidden curriculum, reinforces and promotes the socialization of students in the medical profession [7]. Beside teaching, educators also have to evaluate their students’ professionalism. Approaches to do this are theoretically well-described, yet in practice medical educators experience difficulties when evaluating professionalism [8].

The dominant framework to evaluate professionalism is behaviour-based [6, 9]. Behaviour is the practical, relevant aspect of professionalism through which a learner’s professionalism becomes observable [10–12]. Through their behaviours most medical students show that they gradually develop a professional attitude, but some students display behaviours that raise concerns with their teachers and peer-students [13, 14]. Such behavioural lapses can originate from personal, interpersonal or institutional causes. Discussing these causes among teachers and students can make clear which actions have to be taken, e.g. extra individual guidance for the student, or any other measures at the institutional or organisational level [13].

The evaluation of performance is difficult for several reasons. Firstly, medical educators experience challenges in labelling unprofessional performance. They are reluctant to label students’ behaviours as unprofessional, partly because they do not know which behaviours can be assigned this label [15]. Secondly, educators not only struggle with the uncertainty of the expected standards for students, but also do not know how to articulate their concerns: *what* to document and *how* to document it [3]. As a result educators’ language in assessment forms is vague and indirect [16]. Furthermore, educators are advised to provide behaviour-based comments in formative or summative *In Training Evaluation Reports* [ITERs], but a definition of unprofessional behaviour is lacking [17, 18]. Finally, what is seen as unprofessional is dependent on time and cultural context, which has led to the use of a plethora of terms describing poor professional performance in the medical education literature [19]. All these hurdles complicate the evaluation process, and attribute to a reluctance in denominating unprofessionalism. This results in a lack of supporting documentation for poor performance in assessment forms [3].

As a result of their reluctance in denominating unprofessionalism, educators do not always make students aware of their unprofessional behaviour. Consequently, they miss the opportunity to *explicitly* teach professionalism by revealing underlying causative personal, interpersonal and/or organisational factors. Another result of this reluctance is that by not acknowledging unprofessional behaviour, educators *implicitly* create the impression that this behaviour is acceptable. This way, educators give rise to an undesirable culture [6, 8, 20].

What could help to overcome these difficulties in the evaluating process is a shared mental model across assessors of what a student should be able to do. With clear expectations of desired professional performance, it may be easier for supervisors to report behaviour that does not meet standards. This implies that we also need clear descriptions of what a student is not expected to do. To discover the unprofessional manifestations of desired behaviours, it could be helpful to look at what has been perceived as unprofessional in the lived experience of educators and students. Which terms are used by educators to express their concerns about students’ unprofessionalism? Which themes of unprofessional behaviours are seen by them? [18] A common understanding among educators about the denomination of unprofessional behaviours could lead to a greater consistency in observing, describing and evaluating it.

The current integrative, systematic review study uses the behaviour-based professionalism framework [6, 9]. It aimed to explore, describe and categorise results of studies describing medical students’ unprofessional behaviours, witnessed by stakeholders or admitted by students themselves, to create an overview of descriptors for these behaviours. The research question that guided this review was: Which descriptions are used in medical education research studies to describe medical students’ behaviours that have actually occurred and were identified as unprofessional, and how can we categorise these?

## Methods

### General methodology

We conducted a systematic review, in which content analysis was used, a qualitative method to analyse text-based data, to identify descriptions of unprofessional behaviours of preclinical and clinical medical students, admitted by students or witnessed by stakeholders [21]. We developed a review protocol based on the Preferred Reporting Items for Systematic Reviews and Meta-Analysis [PRISMA]-statement [22]. Due to the diversity of the methodologies in the included articles, we did not perform a meta-analysis. The review protocol is available upon request.

All authors are researchers in medical education. MM, WM, GC and RAK are medical doctors, JK is a midwife. All are experienced in the guidance of students who display unprofessional behaviour. SB is a sociologist and a PhD student in medical education, and JCFK is an information specialist.

### Data sources and search strategy

MM and JCFK systematically searched the databases PubMed, Embase.com, Ebsco/ERIC and Ebsco/PsycINFO from inception to May 2016, using the following search terms as index-terms or free-text words: “medical students” OR “medical education” AND “professional misconduct” OR “malpractice” OR “dishonesty”, and related terms. The complete search strategy can be found in Additional file 1. All languages were included, and duplicate articles excluded. Articles in languages unknown to the authors, were read by a native speaker, who explained the content to the first author.

### Study selection

Articles that described quantitative and/or qualitative original studies reporting witnessed or admitted unprofessional behaviours of preclinical and clinical medical students were eligible for inclusion. In absence of a commonly accepted definition of ‘unprofessional behaviour’, articles were included if the authors described the behaviours as *unprofessional*, or used the descriptions *misconduct*, *malpractice*, *lapse*, *underperformance*, *nonprofessional*, *adverse*, *negative*, *problematic*, *professionalism issues, professionalism dilemmas, professionalism challenges, professionalism problems* or *professionalism concerns*. These terms were chosen based on the literature and the set was finalised in the research team in consensus. Articles were excluded if they described unprofessional behaviours of residents or physicians, or if they described hypothetical behaviours, or behaviours that occurred outside the educational context. Two authors (MM, and either WM, SB, JK, or RAK) independently reviewed each abstract to identify articles that were considered relevant for possible inclusion in the review. In case of doubt, the full article was screened. Disagreements about search terms or eligibility were discussed in the research team until consensus was reached.

### Data extraction and synthesis

Data were extracted using a coding sheet based on the Best Evidence Medical Education (BEME) collaboration [23], including the following BEME coding items: the administrative item, the evaluation methods, and the context. Based on the content analysis review method the following “unit of analysis” was added to the coding sheet: descriptions of medical students’ unprofessional behaviours that were witnessed by stakeholders or admitted by students themselves. Reported findings were extracted onto the coding sheets.

The methodological quality of the articles was assessed by answering the following five quality questions: [i] Is the research question or purpose clearly stated?, [ii] Is the method used suitable for answering the research question?, [iii] Are the methods and results clearly described?, [iv] Is the method of analysis appropriate?, and [v] Is the research question answered by the data? [24] Studies were considered to be of higher quality when more questions could be answered positively.

The first author and one of the co-authors independently performed data extraction, coding, and quality assessment, a third author being involved if necessary to reach consensus. Coding was completed inductively during the analysis. The researchers also drafted written notations about the data during the coding process, the so-called “memos” [21]. The research team reflected as a group on identified codes and memos, and used these as aids in organizing the content, and categorising it into themes. A constant comparative approach was used, meaning that the researchers brought their ideas together in a cyclic process of reading, writing, reflecting and revising. (21) Differences of opinion about quality assessment, data extraction and classification of findings were discussed until consensus was reached.

## Results

### Search results

**Fig. 1:**
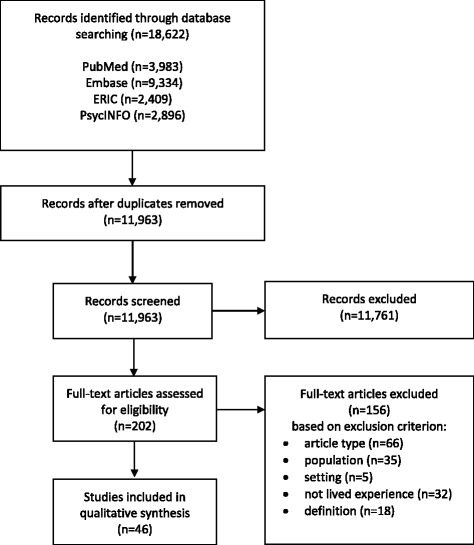
Flow diagram of literature search and study selection

The search yielded 11,963 different articles: 202 were identified as relevant after initial screening of titles and abstracts and 46 were included after reviewing the full texts. See Fig. 1. A list of excluded studies with justifications is provided as Additional file 2.

### Study characteristics

The review included studies from a wide range of countries, from January 1977–May 2016. See additional file 3 for an overview of the 46 included studies. We included 30 quantitative studies, 11 qualitative and 5 mixed-methods studies. Three of the articles were not written in the English language: two were written in Spanish and one in Greek. From the included articles, 29 described single-institution studies and 17 described multi-institution studies, varying from 2 to 78 institutions. In 28 articles a survey was described, and 16 other articles reported case-studies using interviews, essays, or students’ records from the university administration. Two additional articles reported observational studies. From the 46 articles, 29 were of good quality. For some articles not all quality questions could be answered positively due to a low response rate.

**Fig. 2:**
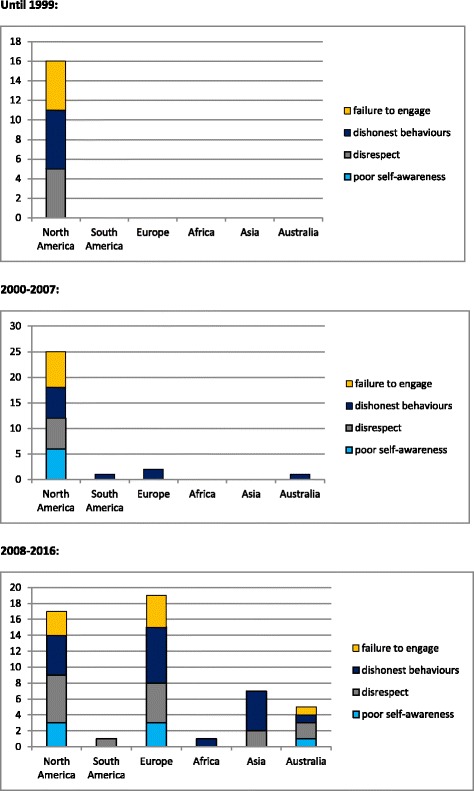
Occurrence of descriptions of behaviours categorised in each of the four themes, in three different time periods

Attention for professional behaviour in medical school started in the US around 1980, firstly emphasised on fraudulent behaviours, followed by attention for disrespectful behaviour and failure to engage. We did not find any articles coming from the other continents that were published before 2000. Around 2000, North-American researchers started to focus on poor self-awareness, while in other continents only dishonest behaviour was described, later followed by other themes. Recently, attention was paid in the literature to unprofessionalism originating from the use of the internet, which can lead to privacy violations and other disrespectful behaviour, as well as to dishonest behaviours. See Fig. 2 for global trends in three time periods.

### Themes of unprofessional behaviour

**Fig. 3:**
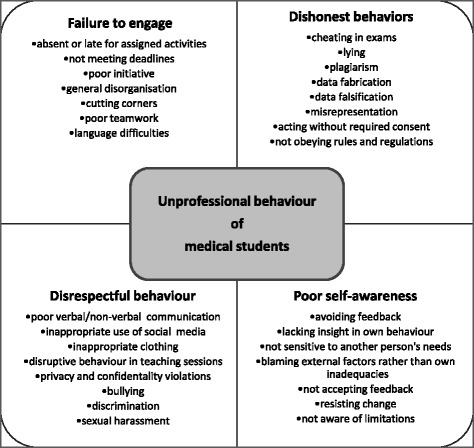
Four themes including 30 descriptors for unprofessional behaviours of medical students

The included articles yielded 205 different descriptions of unprofessional behaviours, which were coded into 30 different descriptors, and subsequently classified into four behavioural themes: *failure to engage*, *dishonest behaviour*, *disrespectful behaviour*, and *poor self-awareness*. See Fig. 3.

The next paragraphs present the primary findings for each of the four themes. See Additional file 4 for a complete and detailed list of themes, descriptors and behaviours.

### Failure to engage

The first theme can be described as *failure to engage*, which was defined as insufficiently handling one’s tasks. *Failure to engage* [25–27] included descriptions as *being late or absent for rounds or other assigned activities* [28–32], *poor reliability and responsibility* [25, 31, 33, 34], *poor availability* [32], *lack of conscientiousness* [35], *tardiness* [32] and *poor initiative and motivation* [31, 32, 36–38], *cutting corners* [39], and *accepting or seeking a minimally acceptable level of performance* [25]. *General disorganization* was mentioned [26, 27], examples of which were *illegible handwriting*, *poor note keeping* and *not meeting deadlines* [32]. Behaviours indicating failure to engage leading to poor teamwork were described as *avoiding work* [27], *escaping teamwork* [40], *language difficulties* [37] and *not giving feedback to others* [30].

Failure to engage in the clinical phase of medical school was seen in the form of *avoidance of patient contact* [27, 37], *failing to contribute to patient care* [26, 37, 39], *leaving the hospital during a shift* [41], and unsatisfactory *participation* [33, 36].

### Dishonest behaviours

This theme describes students’ integrity problems. It includes cheating, lying, plagiarism and not obeying rules and regulations.

### Cheating and lying

Cheating and lying took place in class by *forging signatures* [40, 42, 43], *or giving false excuses when absent* [40, 43–47], *asking a colleague to sign in on an attendance list* [26, 41, 43, 45, 48], *asking other students to do your work* or *doing work for another student* [40, 41, 43]. *Cheating in exams* [32] was extensively described, and consisted of: *gaining illegal access to exam questions* [40, 43–47], *letting someone else take your exam* [43, 46, 47], *using crib notes* [43, 44, 46–49], *exchanging answers during an exam* [43–49], *exchanging answers by using mobile phones* [43, 45, 48] and *passing an exam by using help from acquaintances* [43, 48, 50]. Cheating in clinical or research context took place in the form of *data fabrication* [26, 40, 41, 43–46, 49, 51–53], and *data falsification* [25, 31, 32, 37, 40, 41, 43, 51–54], sometimes to disguise mistakes [43], e.g. when a student had forgotten to order a laboratory test or omitted a part of the history taking or physical examination [40, 41, 44, 46, 49, 51, 55]. Also, *not asking consent for clinical examination of a patient *was mentioned [56, 57]. One study reported cheating in using the hospital’s electronic health record documentation [EHRD]: *copy/pasting a colleague’s notes*, *using auto-inserted data*, or *documenting while signed in under someone else’s name in the EHRD* [58].

Already in 1978, a law scholar, Simpson, emphasised the phenomenon of *deceptive introduction* [59] Students being introduced as “doctors” to patients is a form of lying that directly influences patient care. This type of misrepresentation has also been described more recently [35, 57].

#### Plagiarism

Plagiarism consisted of *self-plagiarism* [43], *plagiarizing work of seniors* or *peers* [46, 52], and *plagiarizing from other sources without acknowledging the reference* [40, 42, 47, 60]. *Copying text directly from published books or articles* was seen as unprofessional even when the source was included in the reference list [43].

#### Not obeying rules and regulations

Unprofessional activities mentioned were: *acceptance of failing to obey rules and regulations* [26] for example by *not following infection control procedures* [43, 57] and *using phones in restricted areas* [61].

Unlicensed activities that were mentioned in the included articles were: *significant misconduct* [32, 42], *stealing* [62], *damaging another’s property* [62] or *physically assaulting a university employee or fellow student* [43].

### Disrespectful behaviour

Another theme was found to be *disrespectful behaviour*, which was defined as behaviour that has a negative effect on other people. Behaviours in this theme vary widely in severity.

Disrespectful behaviour was described as poor verbal or non-verbal communication: *inappropriate spoken language* [25, 26, 32, 56, 63] *inappropriate body language* [26–28, 32], *disrespectful communication by email* [32] and also *ignoring emails or other forms of contact from teaching or administrative staff* [26, 36]. Recent articles mentioned unprofessional behaviour on Facebook or other social media*,* for example d*iscussing clinical experiences with patients* [64] *discussing a clinical site or the university in a negative light* [64] and *posting compromising pictures of peer students* [63, 65]. Other disrespectful behaviours that are exemplary for the lack of sensitivity to others’ needs were *cultural and religious insensitivity* [35], *discrimination* [33, 35], and *sexual harassment* [35, 43, 63]. These disrespectful behaviours can affect all persons with whom these students interact: teachers and other staff or health personnel, patients and their families, or fellow students.

Teachers can be treated disrespectfully by n*egative responses or disruptive behaviour in teaching sessions* [26, 34, 36, 66], writing *rude/inappropriate comments on exam papers* [26] or other *failure to show respect for the examination process* [28].

Patients can be affected by a student’s disrespectful behaviour when the student shows *a lack of empathy* [26, 28], *insensitivity to the needs of others* [25, 26, 62], and *abrupt and non-empathetic manner with patients* [26], *referring to patients in a derogatory way* [29, 30, 39, 56, 57], *placing own learning above patient safety* [57], *making a patient feel uncomfortable during an exam* [56] or *treating simulation patients as passive objects rather than as people with feelings and concerns* [28] were examples of behaviours that were seen as a lack of empathy. Also, overly *informal behaviour* [27], and *failure to maintain professional appearance and attire* [25, 26, 28, 30, 37] and *poor condition of white coats* [29, 30] belong to this theme. Furthermore, *discussing patients in public spaces* [29] and therefore *failing to respect patient confidentiality* [25, 30, 35, 56, 63] or *using Google to research patients* [67] were described as unprofessional.

Fellow students can be treated disrespectfully through *bullying* by peers, which consist of *verbal, written, physical or behavioural abuse* and *victimizing*, which is the *ignoring of someone’s existence* [43, 62, 68, 69]. Students can also be affected by their peers‘ unprofessional behaviour by *reporting a peer’s improper behaviour to faculty before approaching the person individually* [29, 30].

### Poor self-awareness

The last theme is *poor self-awareness*, which was defined as inappropriately handling one’s own performance. Poor self-awareness was described as *avoiding feedback, inability to accept and incorporate feedback* [30, 31, 38], and *resistant or defensive behaviour towards criticism* [25, 34, 37], *lack of insight into behaviour* [26, 28], *blaming external factors rather than own* [28] and *failing to accept responsibility for actions* [25, 28]. Furthermore, *not being aware of limitations* [32], *acting beyond own level of competence* [56, 57], or *not respecting professional boundaries* [26, 63] was categorised in this theme. These behaviours seem to indicate a *diminished capacity for self-improvement* [32, 34, 37, 70].

## Discussion

There is a need for consistent terminology to describe unprofessional behaviours, and therefore the purpose of this systematic review was to create an overview of descriptions of real-life unprofessional behaviours of medical students. Based on the included articles, 205 found descriptions of unprofessional behaviours were summarised as 30 descriptors, and categorised into four themes: *failure to engage*, *dishonest behaviour*, *disrespectful behaviour* and *poor self-awareness*. The descriptors of the behaviours belonging to these themes could prompt medical educators to better recognise, denominate and acknowledge these behaviours in daily practice.

### Search results and study characteristics

Most studies came from a single institution, which often resulted in a limited number of students, and limited diversity in cultural context. Collaboration across institutions and countries would add greatly to the research of unprofessional behaviour.

Professionalism is a concept that varies in time and place, which becomes clear from the subjects that were investigated in the included articles. Surprisingly, the descriptions of behaviours that were seen as unprofessional did not differ largely between the continents, although in Asia and Africa the focus seems to lay on dishonest behaviours. Probably, the research on unprofessional behaviour starts with a focus on fraudulent behaviour because it is seen as a serious problem that is easy to detect. Recently described topics in the medical education literature are self-awareness and reflection, and the person of the doctor him/herself [2, 71]. This trend, representing a more positive approach to unprofessional behaviour, seems to have come over from North America to Europe and Australia, and it will be interesting to see if this trend will spread to South America, Africa and Asia in the coming years.

Only two studies described bullying, while the report of the Expert Advisory Group to the Royal Australasian College of Surgeons describes that the culture of bullying is widespread among physicians [72]. This could either mean that researchers do not pay attention to bullying, or that teachers and students need to be trained in recognizing and reporting bullying.

### Themes of unprofessional behaviour

The behaviours found in this study are specific for students in undergraduate education and have not been described extensively in existing guidelines [73–75]. The themes found in this study resemble the domains from guidelines, although in this study not all guideline domains were found, which indicates that some of these domains seem to be specific for physicians and are not applicable to students.

A recent review revealed that unprofessional behaviours in future physicians are seen in the theme of fraud and dishonest behaviour [76]. The current study extends these findings with three additional themes by including additional articles. This was a result of a broad search strategy using a comprehensive range of terms used in the international literature on unprofessional behaviour, and inclusion of quantitative as well qualitative studies.

Previous research proposed six domains in which evidence of professionalism can be expected from doctors-in-training: *responsibility for actions*, *ethical practice*, *respect for patients*, *reflection/self-awareness*, *teamwork*, and *social responsibility* [77]. Current findings are partly consistent with this framework, although only four themes were distinguished. Examples of students’ behaviours that can be regarded as *poor social responsibility* were not found. This domain might be more relevant for residents than for undergraduate students. Furthermore, from this study *poor teamwork* seems to be a result of behaviours that indicate a failure to engage. The currently found behaviours can be seen as a practical addition to this framework.

The General Medical Council (GMC) recently published an updated professionalism guidance for medical students, in which domains of concern are described [2]. We mapped our findings to these normative descriptions and found many similarities, but also some differences. We did not find concerns that indicate a cause for unprofessional behaviour, such as drug abuse, since we searched for behaviours that teachers would see in the educational environment, and not for underlying causes. Our findings add to the GMC domains by including some new descriptors. Additional file 5 shows in detail how our findings were mapped to the GMC’s domains of concern.

Engagement, integrity, respect and self-awareness matter in medical school, as they do in physician life. By exhibiting these behaviours students can gain trust of faculty and peers, just as doctors gain trust of colleagues and patients. A crucial question is whether the behaviours found in students relate to future unprofessional behaviours as a physician. This has been shown for *poor initiative*, *irresponsibility* and *diminished capacity for self-improvement*, but it is not yet known whether the other behaviours found in this study also predict future performance as a physician [25, 70].

### Failure to engage

When poor engagement is a consequence of physical or mental illness, students have to be supported in acknowledging this, and offered possibilities to continue and complete their studies [78]. Engagement problems related to the quality and quantity of student motivation could be addressed by using Self-determination Theory, which offers possibilities to enhance engagement by fostering student motivation by paying attention to three key elements: autonomy, relatedness and competence of the learners [79]. This method has been described in twelve practical tips that medical educators can apply in class [80].

### Dishonest behaviour

Dishonest behaviours are rarely isolated events and individuals involved in cheating are more likely to be involved in other dishonest behaviours [81]. Failing to complete required course evaluations and failing to report immunization compliance were found to be significant predictors of students’ unprofessional behaviours in subsequent years [82]. Thus, it seems necessary to raise faculty’s awareness for students not obeying rules and regulations and committing dishonest behaviours [52]. Software to detect plagiarism can help to unveil some of these behaviours [83].

### Disrespectful behaviour

Although disrespectful behaviour might be experienced differently in different time periods and in different parts of the world, the terms that are used to describe disrespectful behaviour are surprisingly consistent over time and place.

Disrespect towards colleagues inhibits collegiality and teamwork, and disrespect towards patients inhibits empathic relations with patients [84]. Disrespectful behaviour, of which bullying and racism are extreme examples, is often tolerated and even reinforced by others [85]. As disrespect is mostly a learned behaviour, it is possible to tackle it with positive role modeling and formal education [85]. However, unfortunately, students are sometimes exposed to very negative and problematic role models who at times are disrespectful [86]. Fear of retaliation can lead a student to act unprofessionally him/herself too [87]. Students should have the opportunity to report unprofessional behaviour of their teachers and supervisors to the school management. Furthermore, educational interventions to maintain and enhance empathy in medical students could be applied [88].

Compromising privacy is also a form of disrespectful behaviour. According to this study, new challenges for maintaining privacy of patients, but also of students and physicians, come from the use of digital media and electronic health record documentation systems. Professionalism is a dynamic concept [89], and it seems that new values and standards for students as well as for physicians have to be developed regarding “digital professionalism.” [90–92].

### Poor self-awareness

Behaviours in this theme are displayed by students who are insufficiently aware of their own poor performance: the student thinks to perform better than the external evaluation indicates. If we want to measure insight, reflective ability and capacity to change, we have to combine different measurements to come to a judgment [93]. A diminished reflective ability is related to professionalism lapses [94], and forms a challenge for remediation, since insight into one’s behaviour is regarded necessary to change it [82, 95]. For students struggling with this aspect of professionalism, educators need to clearly set expectations based on the performance of peers [96].

### Context of unprofessional behaviour

Personal, interpersonal and institutional circumstances have to be taken into account when evaluating a student’s professional behaviour [97, 98]. This list of behaviours indicates which behaviours should be a reason to have a discussion with the student, aiming for an interpretation in the context that could reveal if the behaviour was indeed unprofessional. Since we want to prepare students for a challenging work environment, it is crucial to teach students how to effectively handle certain difficult contextual conditions that are likely to happen in their future work, like unprofessional behaviours of others, stressful conditions and time constraints [3, 84, 99]. Students and teachers have to discuss and negotiate what behaviours could be adequate in difficult circumstances. Role modeling is not enough; formal teaching when these difficult conditions occur [in the clerkships] is deemed necessary [100].

### Limitations

The terminology that is used in the literature on professionalism varies widely. A broad range of search terms was applied, restricted to negatively formulated terms based on admitted or witnessed behaviours by stakeholders. A limitation of this method is that there may be some unprofessional behaviours which go unrecognised or unreported by teachers and students. These -still hidden- behaviours might be revealed when speaking about lapses becomes more commonly accepted using the terminology that we propose.

Some relevant articles could not be included because the researchers used an integrated description of behaviours of students, faculty and physicians from which the students’ behaviours could not be separated [90, 98]. However, after checking, it was verified that including these articles would not have changed the results.

We aimed to describe real-life behaviours, and chose to use content analysis of research articles to capture these. Consequently, our method could not reveal behaviours that were not described in research articles. It has to be acknowledged that potentially some parts of the world are underrepresented due to the limited number of original research papers originating from some regions, which consequently could have led to an underreporting of certain behaviours.

Furthermore, generalizations in this review are based on a wide variety of types of studies, coming from different parts of the world and from different time periods. Although we designed the review purposefully in this way, we acknowledge that the differences in study design and participating stakeholders might limit the generalizability of the results. Further research should reveal the applicability of the proposed framework in different contexts.

### Practical implications

The results of this review provide medical educators and researchers in medical education with a common language for the description of unprofessional behaviour in preclinical and clinical undergraduate medical education. Knowledge of the nature and extent of students’ unprofessional behaviours could prompt teachers, and facilitate the acknowledgment and discussion of these behaviours among teachers and students. The list might facilitate teachers to see and report unprofessional behaviours, and thus help to solve the problem of “failure to fail”. Yet, only giving a fail is not enough: it is necessary that educators conduct a conversation with the student about observed behaviours. Such a conversation, in which explanations are given and context is discussed, can lead to a fair assessment and to a valuable formative learning experience for the student, or to other actions needed to improve interpersonal or institutional causes for unprofessional behaviour [101, 102] (Table 1).

**Table 1:** Descriptors for unprofessional behaviours of medical students: a systematic review and categorisation

Implications	
Common language	Facilitates the acknowledgment and discussion of unprofessional behaviours among teachers and students
	Could prompt researchers to reach agreement about descriptors as common ground for research
List of unprofessional behaviours	Facilitates teachers to see and report unprofessional behaviours
	Could add to existent frameworks on professionalism

### Further research

Further action is desirable to reach consensus among stakeholders all over the world to endorse language as proposed in this study, and reach agreement about descriptors for unprofessional behaviours. A common language is needed not only for teaching, assessment and remediation, but also to provide a common ground for further research.

This study addressed one reason for educator’s reluctance to fail students, but other reasons require further exploration as well. Furthermore, research about remediation of unprofessional behaviour is deemed necessary [103]. Failure to engage could be related to insufficient student motivation. Empirical study of this issue might generate interesting findings, especially because student motivation is dynamic and can be influenced [79].

Another subject that needs investigation is students’ accountability for their peers. Recently, a US nation-wide study found that a significant majority of students said that they feel obligated to report unprofessional behaviour of their peers [104]. This leads to the question: How can we educate these students to change their intentions into actions?

## Conclusions

Descriptors for 30 unprofessional behaviours have been categorised in four themes: *failure to engage*, *dishonest behaviour*, *disrespectful behaviour *and *poor self-awareness.* In medical school these behaviours have to be acknowledged, addressed, evaluated, and discussed between students and teachers. This is beneficial for *all* students: students who behaved unprofessionally can profit from timely offered remediation, and students with satisfactory professional behaviour will learn how to respond to unprofessional behaviour when they see their teachers take these problems seriously. Such a policy would contribute to a culture of professionalism excellence, which is ultimately beneficial for all stakeholders, including patients.

## Additional files

Additional file 1: Complete search strategy. (DOCX 26 kb)

Additional file 2: Excluded articles with justification. (RTF 531 kb)

Additional file 3: Included 46 studies. (RTF 530 kb)

Additional file 4: List of themes, descriptors and behaviours. (DOCX 40 kb)

Additional file 5: Findings mapped to GMC’s domains of concern. (DOCX 30 kb)
