# Supplementary material for: Descriptors for unprofessional behaviours of medical students: a systematic review and categorisation
Source: BMC Med Educ. 2017 Sep 15;17:164. doi: 10.1186/s12909-017-0997-x (PMC5603020; doi:10.1186/s12909-017-0997-x)
Supplement: Supplementary file 1 — Complete search strategy. (DOCX 26 kb) [file 12909_2017_997_MOESM1_ESM.docx]

**Embase.com May 9, 2016**

| **No.** | **Query** | **Results** |
| --- | --- | --- |
| #4 | **#3** NOT [medline]/lim | **3161** |
| #3 | **#1** AND **#2** | **9334** |
| #2 | **'misconduct'**/exp OR **'malpractice'**/exp OR **'sexual harassment'**/exp OR **'social discrimination'**/exp OR **'bullying'**/exp OR (**unprofessional***:ab,ti OR **adverse**:ab,ti OR **disrupti***:ab,ti OR **worrying**:ab,ti OR **inappropria***:ab,ti OR **unethical***:ab,ti OR **irregular***:ab,ti OR **problematic***:ab,ti OR **hostile**:ab,ti OR **immoral***:ab,ti OR **nonprofessional***:ab,ti OR (**self** NEAR/3 **absorb***):ab,ti OR **arrogant***:ab,ti OR **'self driven'**:ab,ti OR **inadequat***:ab,ti OR **dishonest***:ab,ti OR **disrespect***:ab,ti OR **irresponsibl***:ab,ti OR **immatur***:ab,ti AND (**conduct***:ab,ti OR **autonom***:ab,ti OR **competenc***:ab,ti OR **performanc***:ab,ti OR **behavi***:ab,ti OR **engagement***:ab,ti OR **lapse***:ab,ti OR **behavior***:ab,ti OR **behaviour***:ab,ti OR **act**:ab,ti OR **acts**:ab,ti OR **actions**:ab,ti)) OR **misconduct***:ab,ti OR **misbehavio***:ab,ti OR **underperform***:ab,ti OR **falsif***:ab,ti OR **fraud***:ab,ti OR **plagiar***:ab,ti OR **probit***:ab,ti OR **fabricat***:ab,ti OR **disintegrit***:ab,ti OR **unprofessionalism***:ab,ti OR **hostility**:ab,ti OR **impair***:ab,ti OR **discompeten***:ab,ti OR **incompeten***:ab,ti OR **unreliab***:ab,ti OR **independabl***:ab,ti OR **untrustworth***:ab,ti OR **discriminati***:ab,ti OR **racism***:ab,ti OR **racist***:ab,ti OR **sexism***:ab,ti OR **lying**:ab,ti OR **cheating**:ab,ti OR **misrepresentati***:ab,ti OR **indebtedness***:ab,ti OR (**boundary** NEAR/3 **violation***):ab,ti OR **'failure to engage'**:ab,ti OR (**poor** NEAR/3 **organisation***):ab,ti OR (**poor** NEAR/3 **organization***):ab,ti OR **disorgani***:ab,ti OR **'lack of insight'**:ab,ti OR **'lack of insights'**:ab,ti OR (**professional*** NEAR/3 (**lapse*** OR **dilemma*** OR **narrative*** OR **difficult*** OR **barrier*** OR **challenge***)):ab,ti OR (**'discourses of'** NEAR/3 **professional***):ab,ti | **1218169** |
| #1 | **'medical education'**/de OR **'medical school'**/exp OR **'clinical education'**/exp OR **'teaching round'**/exp OR **intern**:ab,ti OR **interns**:ab,ti OR **internship**:ab,ti OR **trainee***:ab,ti OR **clerk***:ab,ti OR (**medical** NEAR/3 **student***):ab,ti OR (**premedical** NEAR/3 **student***):ab,ti OR (**medical** NEAR/3 **school***):ab,ti OR (**premedical** NEAR/3 **school***):ab,ti OR (**future** NEAR/3 **physician***):ab,ti | **282181** |

**PubMed May 9, 2016**

| **Search** | **Query** | **Items found** |
| --- | --- | --- |
| [#5](http://www.ncbi.nlm.nih.gov/pubmed/advanced) | (#3 NOT #4) | [48](http://www.ncbi.nlm.nih.gov/pubmed/?cmd=HistorySearch&querykey=5) |
| [#4](http://www.ncbi.nlm.nih.gov/pubmed/advanced) | ((("Education, Medical"[Mesh:noexp] OR "Education, Medical, Undergraduate"[Mesh] OR intern[tiab] OR interns[tiab] OR internship[tiab] OR trainee*[tiab] OR clerk*[tiab] OR medical student*[tiab] OR premedical student*[tiab] OR medical school*[tiab] OR premedical school*[tiab] OR future physician*[tiab] OR intern[ot] OR interns[ot] OR internship[ot] OR trainee*[ot] OR clerk*[ot] OR medical student*[ot] OR medical school*[ot] OR premedical school*[ot] OR future physician*[ot] OR premedical student*[ot]) AND ("Professional Misconduct"[Mesh] OR "Professional Impairment"[Mesh] OR "Malpractice"[Mesh] OR "Plagiarism"[Mesh] OR "Sexual Harassment"[Mesh] OR "Social Discrimination"[Mesh] OR "Bullying"[Mesh] OR ((unprofessional*[tiab] OR adverse[tiab] OR disrupti*[tiab] OR worrying[tiab] OR inappropria*[tiab] OR unethical*[tiab] OR irregular*[tiab] OR immoral*[tiab] OR problematic*[tiab] OR hostile[tiab] OR immoral*[tiab] OR dishonest*[tiab] OR nonprofessional*[tiab] OR (self[tiab] AND absorb*[tiab]) OR arrogant*[tiab] OR "self driven"[tiab] OR inadequat*[tiab] OR dishonest*[tiab] OR disrespect*[tiab] OR irresponsibl*[tiab] OR immatur*[tiab]) AND (conduct*[tiab] OR autonom*[tiab] OR competenc*[tiab] OR performanc*[tiab] OR behavi*[tiab] OR engagement*[tiab] OR lapse* OR behavior*[tiab] OR behaviour*[tiab] OR act[tiab] OR acts[tiab] OR actions[tiab])) OR misconduct*[tiab] OR misbehavio*[tiab] OR underperform*[tiab] OR falsif*[tiab] OR fraud*[tiab] OR plagiar*[tiab] OR probit*[tiab] OR fabricat*[tiab] OR disintegrit*[tiab] OR unprofessionalism*[tiab] OR hostility[tiab] OR impair*[tiab] OR discompeten*[tiab] OR incompeten*[tiab] OR unreliab*[tiab] OR independabl*[tiab] OR untrustworth*[tiab] OR discriminati*[tiab] OR racism*[tiab] OR racist* OR sexism*[tiab] OR lying[tiab] OR cheating[tiab] OR misrepresentati*[tiab] OR indebtedness*[tiab] OR boundary violation*[tiab] OR failure to engage*[tiab] OR (poor[tiab] AND organisation*[tiab]) OR (poor[tiab] AND organization*[tiab]) OR disorgani*[tiab] OR lack of insight*[tiab] OR ((unprofessional*[ot] OR adverse[ot] OR disrupti*[ot] OR worrying[ot] OR inappropria*[ot] OR unethical*[ot] OR irregular*[ot] OR immoral*[ot] OR problematic*[ot] OR hostile[ot] OR immoral*[ot] OR dishonest*[ot] OR nonprofessional*[ot] OR (self[ot] AND absorb*[ot]) OR arrogant*[ot] OR "self driven"[ot] OR inadequat*[ot] OR dishonest*[ot] OR disrespect*[ot] OR irresponsibl*[ot] OR immatur*[ot]) AND (conduct*[ot] OR autonom*[ot] OR competenc*[ot] OR performanc*[ot] OR behavi*[ot] OR engagement*[ot] OR lapse* OR behavior*[ot] OR behaviour*[ot] OR act[ot] OR acts[ot] OR actions[ot])) OR misconduct*[ot] OR misbehavio*[ot] OR underperform*[ot] OR falsif*[ot] OR fraud*[ot] OR plagiar*[ot] OR probit*[ot] OR fabricat*[ot] OR disintegrit*[ot] OR unprofessionalism*[ot] OR hostility[ot] OR impair*[ot] OR discompeten*[ot] OR incompeten*[ot] OR unreliab*[ot] OR independabl*[ot] OR untrustworth*[ot] OR discriminati*[ot] OR racism*[ot] OR racist* OR sexism*[ot] OR lying[ot] OR cheating[ot] OR misrepresentati*[ot] OR indebtedness*[ot] OR boundary violation*[ot] OR failure to engage*[ot] OR (poor[ot] AND organisation*[ot]) OR (poor[ot] AND organization*[ot]) OR disorgani*[ot] OR lack of insight*[ot]))) | [4080](http://www.ncbi.nlm.nih.gov/pubmed/?cmd=HistorySearch&querykey=4) |
| [#3](http://www.ncbi.nlm.nih.gov/pubmed/advanced) | (#1 AND #2) | [3983](http://www.ncbi.nlm.nih.gov/pubmed/?cmd=HistorySearch&querykey=3) |
| [#2](http://www.ncbi.nlm.nih.gov/pubmed/advanced) | "Professional Misconduct"[Mesh] OR "Professional Impairment"[Mesh] OR "Malpractice"[Mesh] OR "Plagiarism"[Mesh] OR "Sexual Harassment"[Mesh] OR "Social Discrimination"[Mesh] OR "Bullying"[Mesh] OR ((unprofessional*[tiab] OR adverse[tiab] OR disrupti*[tiab] OR worrying[tiab] OR inappropria*[tiab] OR unethical*[tiab] OR irregular*[tiab] OR immoral*[tiab] OR problematic*[tiab] OR hostile[tiab] OR immoral*[tiab] OR dishonest*[tiab] OR nonprofessional*[tiab] OR (self[tiab] AND absorb*[tiab]) OR arrogant*[tiab] OR “self driven”[tiab] OR inadequat*[tiab] OR dishonest*[tiab] OR irresponsibl*[tiab] OR immatur*[tiab] OR disrespect*[tiab]) AND (conduct*[tiab] OR autonom*[tiab] OR competenc*[tiab] OR performanc*[tiab] OR behavi*[tiab] OR engagement*[tiab] OR lapse*[tiab] OR behavior*[tiab] OR behaviour*[tiab] OR act[tiab] OR acts[tiab] OR actions[tiab])) OR misconduct*[tiab] OR misbehavio*[tiab] OR underperform*[tiab] OR falsif*[tiab] OR fraud*[tiab] OR plagiar*[tiab] OR probit*[tiab] OR fabricat*[tiab] OR disintegrit*[tiab] OR unprofessionalism*[tiab] OR hostility[tiab] OR impair*[tiab] OR discompeten*[tiab] OR incompeten*[tiab] OR unreliab*[tiab] OR independabl*[tiab] OR untrustworth*[tiab] OR discriminati*[tiab] OR racism*[tiab] OR racist* OR sexism*[tiab] OR lying[tiab] OR cheating[tiab] OR misrepresentati*[tiab] OR indebtedness*[tiab] OR boundary violation*[tiab] OR failure to engage*[tiab] OR (poor[tiab] AND organisation*[tiab]) OR (poor[tiab] AND organization*[tiab]) OR disorgani*[tiab] OR lack of insight*[tiab] OR professional lapse*[tiab] OR professionalism lapse*[tiab] OR professional dilemma*[tiab] OR professional narrative*[tiab] OR professionalism dilemma*[tiab] OR professionalism narrative*[tiab] OR professional difficult*[tiab] OR professional barrier*[tiab] OR discourses of professional*[tiab] OR professional challenge*[tiab] | [984976](http://www.ncbi.nlm.nih.gov/pubmed/?cmd=HistorySearch&querykey=2) |
| [#1](http://www.ncbi.nlm.nih.gov/pubmed/advanced) | "Education, Medical"[Mesh:NoExp] OR intern[tiab] OR interns[tiab] OR internship[tiab] OR trainee*[tiab] OR clerk*[tiab] OR medical student*[tiab] OR premedical student*[tiab] OR medical school*[tiab] OR premedical school*[tiab] OR future physician*[tiab] | [113086](http://www.ncbi.nlm.nih.gov/pubmed/?cmd=HistorySearch&querykey=1) |

**Ebsco/PsycInfo May 11, 2016**

| **#** | **Query** | **Results** |
| --- | --- | --- |
| **S3** | S1 AND S2 | **2,896** |
| **S2** | ( DE "Cheating" OR DE "Dishonesty" OR DE "Fraud" OR DE "Resistance" OR DE "Assertiveness" OR DE "Avoidance" OR DE "School Refusal" OR DE "Vandalism" OR DE "Responsibility" OR DE "Accountability" OR DE "Social Responsibility" OR DE "Conscientiousness" OR DE "Professional Liability" OR DE "Sexual Harassment" OR DE "Social Discrimination" OR DE "Age Discrimination" OR DE "Race and Ethnic Discrimination" OR DE "Sex Discrimination" OR DE "Bullying" OR DE "Cyberbullying" OR DE "Relational Aggression" OR DE "Dominance" OR DE "Dominance Hierarchy" OR DE "Abuse of Power" ) OR TI ( ((unprofessional* OR adverse OR disrupti* OR worrying OR inappropria* OR unethical* OR irregular* OR problematic* OR hostile OR immoral* OR nonprofessional* OR (self N3 absorb*) OR arrogant* OR “self driven” OR inadequat* OR dishonest* OR disrespect* OR irresponsib* OR immatur*) AND (conduct* OR autonom* OR competenc* OR performanc* OR behavi* OR engagement* OR lapse* OR behavior* OR behaviour* OR act OR acts OR actions)) OR misconduct* OR misbehavio* OR underperform* OR falsif* OR fraud* OR plagiar* OR probit* OR fabricat* OR disintegrit* OR unprofessionalism* OR hostility OR impair* OR discompeten* OR incompeten* OR unreliab* OR independabl* OR untrustworth* OR discriminati* OR racism* OR racist* OR sexism* OR lying OR cheating OR misrepresentati* OR indebtedness* OR (boundary N3 violation*) OR “failure to engage” OR (poor N3 organisation*) OR (poor N3 organization*) OR disorgani* OR “lack of insight” OR “lack of insights” OR (professional* N3 (dilemma* OR narrative* OR difficult* OR barrier* OR challenge*)) OR (“discourses of” N3 professional*) ) OR AB ( ((unprofessional* OR adverse OR disrupti* OR worrying OR inappropria* OR unethical* OR irregular* OR problematic* OR hostile OR immoral* OR nonprofessional* OR (self N3 absorb*) OR arrogant* OR “self driven” OR inadequat* OR dishonest* OR disrespect* OR irresponsib* OR immatur*) AND (conduct* OR autonom* OR competenc* OR performanc* OR behavi* OR engagement* OR lapse* OR behavior* OR behaviour* OR act OR acts OR actions)) OR misconduct* OR misbehavio* OR underperform* OR falsif* OR fraud* OR plagiar* OR probit* OR fabricat* OR disintegrit* OR unprofessionalism* OR hostility OR impair* OR discompeten* OR incompeten* OR unreliab* OR independabl* OR untrustworth* OR discriminati* OR racism* OR racist* OR sexism* OR lying OR cheating OR misrepresentati* OR indebtedness* OR (boundary N3 violation*) OR “failure to engage” OR (poor N3 organisation*) OR (poor N3 organization*) OR disorgani* OR “lack of insight” OR “lack of insights” OR (professional* N3 (dilemma* OR narrative* OR difficult* OR barrier* OR challenge*)) OR (“discourses of” N3 professional*) ) | **386,060** |
| **S1** | ( DE "Medical Education" OR DE "Medical Internship" OR DE "Medical Residency" OR DE "Psychiatric Training" OR DE "Medical Students" ) OR TI ( intern OR interns OR internship OR trainee* OR clerk* OR (medical N3 student*) OR (premedical N3 student*) OR (medical N3 school*) OR (premedical N3 school*) OR (future N3 physician*) ) OR AB ( intern OR interns OR internship OR trainee* OR clerk* OR (medical N3 student*) OR (premedical N3 student*) OR (medical N3 school*) OR (premedical N3 school*) OR (future N3 physician*) ) | **46,667** |

**Ebsco/ERIC May 11, 2016**

| **#** | **Query** | **Results** |
| --- | --- | --- |
| **S3** | S1 AND S2 | **2,409** |
| **S2** | DE "Cheating" OR DE "Plagiarism" OR DE "Discipline Problems" OR DE "Resistance (Psychology)" OR DE "Resistance to Change" OR DE "Vandalism" OR DE "School Vandalism" OR DE "Computer Security" OR DE "Crime" OR DE "Delinquency" OR DE "Accountability" OR DE "Responsibility" OR DE "Accountability" OR DE "Administrator Responsibility" OR DE "Child Responsibility" OR DE "Community Responsibility" OR DE "Educational Responsibility" OR DE "Leadership Responsibility" OR DE "Legal Responsibility" OR DE "Parent Responsibility" OR DE "School Responsibility" OR DE "Social Responsibility" OR DE "Student Responsibility" OR DE "Teacher Responsibility" OR DE "Sexual Harassment" OR DE "Social Discrimination" OR DE "Age Discrimination" OR DE "Disability Discrimination" OR DE "Educational Discrimination" OR DE "Gender Discrimination" OR DE "Racial Discrimination" OR DE "Religious Discrimination" OR DE "Reverse Discrimination" OR DE "Age Discrimination" OR DE "Disability Discrimination" OR DE "Educational Discrimination" OR DE "School Segregation" OR DE "Gender Discrimination" OR DE "Sexual Harassment" OR DE "Racial Discrimination" OR DE "Racial Segregation" OR DE "Religious Discrimination" OR DE "Reverse Discrimination" OR DE "Bullying" OR TI (((unprofessional* OR adverse OR disrupti* OR worrying OR inappropria* OR unethical* OR irregular* OR immoral* OR problematic* OR hostile OR immoral* OR dishonest* OR nonprofessional* OR (self AND absorb*) OR arrogant* OR “self driven” OR inadequat* OR dishonest* OR disrespect* OR irresponsibl* OR immatur*) AND (conduct* OR autonom* OR competenc* OR performanc* OR behavi* OR engagement* OR lapse* OR behavior* OR behaviour* OR act OR acts OR actions)) OR misconduct* OR misbehavio* OR underperform* OR falsif* OR fraud* OR plagiar* OR probit* OR fabricat* OR disintegrit* OR unprofessionalism* OR hostility OR impair* OR discompeten* OR incompeten* OR unreliab* OR independabl* OR untrustworth* OR discriminati* OR racism* OR racist* OR sexism* OR lying OR cheating OR misrepresentati* OR indebtedness* OR (boundary AND violation*) OR “failure to engage” OR (poor AND organisation*) OR (poor AND organization*) OR disorgani* OR “lack of insight” OR “lack of insights”) OR AB (((unprofessional* OR adverse OR disrupti* OR worrying OR inappropria* OR unethical* OR irregular* OR immoral* OR problematic* OR hostile OR immoral* OR dishonest* OR nonprofessional* OR (self AND absorb*) OR arrogant* OR “self driven” OR inadequat* OR dishonest* OR disrespect* OR irresponsibl* OR immatur*) AND (conduct* OR autonom* OR competenc* OR performanc* OR behavi* OR engagement* OR lapse* OR behavior* OR behaviour* OR act OR acts OR actions)) OR misconduct* OR misbehavio* OR underperform* OR falsif* OR fraud* OR plagiar* OR probit* OR fabricat* OR unprofessionalism* OR hostility OR impair* OR discompeten* OR incompeten* OR unreliab* OR independabl* OR untrustworth* OR discriminati* OR racism* OR racist* OR sexism* OR lying OR cheating OR misrepresentati* OR indebtedness* OR (boundary AND violation*) OR “failure to engage” OR (poor AND organisation*) OR (poor AND organization*) OR disorgani* OR “lack of insight” OR “lack of insights” OR (professional* N3 (dilemma* OR narrative* OR difficult* OR barrier* OR challenge*)) OR (“discourses of” N3 professional*) | **153,061** |
| **S1** | DE "Medical Education" OR DE "Medical Schools" OR DE "Medical Students" OR TI (intern OR interns OR internship OR trainee* OR clerk* OR (medical AND student*) OR (premedical AND student*) OR (medical AND school*) OR (premedical AND school*) OR (future AND physician*) ) OR AB (intern OR interns OR internship OR trainee* OR clerk* OR (medical AND student*) OR (premedical AND student*) OR (medical AND school*) OR (premedical AND school*) OR (future AND physician*) ) | **31,055** |
