# Supplementary material for: Descriptors for unprofessional behaviours of medical students: a systematic review and categorisation
Source: BMC Med Educ. 2017 Sep 15;17:164. doi: 10.1186/s12909-017-0997-x (PMC5603020; doi:10.1186/s12909-017-0997-x)
Supplement: Supplementary file 2 — Excluded articles with justification. (RTF 531 kb) [file 12909_2017_997_MOESM2_ESM.rtf]

Descriptors for unprofessional behaviours of medical students: a systematic review and categorisation
Additional file 2
Excluded articles with justifications 
 

 Title	Exclusion criterion 
	Comments	
Annane D, Annane F. [Plagiarism in medical schools, and its prevention]. Presse Med 2012;41:821-826.	not lived experience	No actual behaviours as outcomes 	
Arora VM, Wayne DB, Anderson RA et al. Changes in perception of and participation in unprofessional behaviors during internship. Acad Med 2010;85:S76-S80.	population	Residents	
Ashworth AJ. Regulating medical students. Misconduct is a behavior, not a state. BMJ 2010;340:c2852	not lived experience	No actual behaviours mentioned	
Babelli S, Chandratilake M, Roff S. Recommended sanctions for lapses in professionalism by student and faculty respondents to Dundee Polyprofessionalism Inventory I: Academic integrity in one medical school in Saudi Arabia. Medical Teacher 2015 Feb;37(2):162-7.	not lived experience	Recommendad sanctions for professionalism lapses	
Bateman J, Francis R, Thistlethwaite J. Medical student burnout and professionalism. JAMA 2011;305:37-38.	article type	Letter, no behaviours mentioned	
Beagan BL. 'Is this worth getting into a big fuss over?' Everyday racism in medical school. Medical Education 2003;37:852-860.	definition	Authors do not speak about unprofessional behaviour	
Bennett AJ, Roman B, Arnold LM, Kay J, Goldenhar LM. Professionalism Deficits among Medical Students: Models of Identification and Intervention. Academic Psychiatry 2005;29:426-432.	not lived experience	No observed behavours	
Bennett-Richards P. Cheating at medical school. Are we all cheats? BMJ 2001;322:298	article type	Letter
Not about actual behaviours	
Beran TN, McLaughlin K, Al Ansari A, Kassam A. Conformity of behaviors among medical students: Impact on performance of knee arthrocentesis in simulation. Advances in Health Sciences Education 2013;18:589-596.	definition	Not about unprofessional behaviour	
Berk R. Derogatory and cynical humour in clinical teaching and the workplace: The need for professionalism. Medical Education 2009;43:7-9.	article type	No research 	
Bernard AW, Malone M, Kman NE, Caterino JM, Khandelwal S. Medical student professionalism narratives: a thematic analysis and interdisciplinary comparative investigation. BMC Emerg Med 2011;11:11.	population	not describing student behaviour	
Bonke B. Unprofessional or problematic behavior of medical students outside the learning environment. Med Teach 2006;28:440-442.	article type	Exclude as it is viewpoint paper and not an empirical article.	
Brewer B. Cheating at medical school. Incident was dealt with appropriately. BMJ 2001;322:296.	article type	Letter, No actual unprof behaviour described
	
Brockbank S, David TJ, Patel L. Unprofessional behavior in medical students: A questionnaire-based pilot study comparing perceptions of the public with medical students and doctors. Medical Teacher 2011;33:e501-e508.	not lived experience	HYPOTHETICAL examples of medical student misconduct	
Brody H. Professionalism versus anti-harassment in student evaluation. Virtual Mentor 2014;16:165-168.	article type	review	
Brooks MH, Watts GT, Thompson JA, Richardson R, Leavitt FJ. Cheating in medical school [10]. Br Med J 1995;311:193-194.	article type	Letter reacting on Leavitt 2015 (not included) Letter
Not about students behaviours	
Byrne A, McKimm J, Jewitt H, Bodger O, Brown M. Multi-source feedback of student professionalism. Med Educ Suppl 2010;44:23.	article type	congress abstract 	
Choi D, Tolova V, Socha E, Samenow CP. Substance Use and Attitudes on Professional Conduct among Medical Students: A Single-Institution Study. Academic Psychiatry 2013;37:191-195	setting 	alcohol abuse, not school behaviours	
Chretien KC, Tuck MG. Online professionalism: A synthetic review. International Review of Psychiatry 2015 Apr;27(2):106-17.	article type	review	
Clark DC. Alcohol and drug use and mood disorders among medical students: implications for physician impairment. QRB Qual Rev Bull 1988;14:50-54.	setting	alcohol abuse, not school behaviours	
Costa MJ, GoncÂ¸alves E, Kislaya I, GoncÂ¸alves M, Salgueira A, Portela M. A longitudinal study identifies early scores in attitudinal dimensions as predictors of students' poor professionalism at the bedside. Med Educ Suppl 2011;45:46.	article type	Congress abstract 	
Cripe LD, Hedrick DG, Rand KL, Burns D, Banno D, Cottingham A, et al. Medical Students' Professionalism Narratives Reveal That Experiences With Death, Dying, or Palliative Care Are More Positive Than Other Experiences During Their Internal Medicine Clerkship. Am J Hosp Palliat Care 2015 Sep 30.	population	Not about medical students 	
Davies S. Cheating at medical school. Summary of rapid responses. BMJ 2001;322:299.	article type	Letter, no actual behaviours mentioned	
Davis WR. More on malpractice concerns in the medical school classroom. N Engl J Med 1986;315:265.	article type	Letter, reaction on Siden 
No behaviours mentioned	
Desalegn AA, Berhan A. Cheating on examinations and its predictors among undergraduate students at Hawassa University College of Medicine and Health Science, Hawassa, Ethiopia. BMC Med Educ 2014;14:89.	population	Not exclusively about medical students	
Dyrbye LN, Harper W, Moutier C et al. A multi-institutional study exploring the impact of positive mental health on medical studentsâ€™ professionalism in an era of high burnout. Academic Medicine 2012;87:1024-1031	not lived experience	response to questionnaire on previously in the literature reported unprofessional behaviours. This providers insight into prevalence but does not add new behaviours	
Fergusson N. Cheating at medical school. Cheating should be properly punished. BMJ 2001;322:297.	article type	Letter
No actual behaviours	
Flaherty JA, Richman JA. Substance use and addiction among medical students, residents, and physicians. Psychiatr Clin North Am 1993;16:189-197.	article type	Flaherty and Richman 1993 - Exclude as it is a literature review and not empirical.	
Galukande M. Students whose behavior causes concern: Am I my brother's keeper? BMJ 2008;337:a2875.	article type	Referring to a letter from students to the BMJ in which they described behaviour form a fellow student , No actual unprof behaviour described 	
Ghias K, Lakho GR, Asim H, Azam IS, Saeed SA. Self-reported attitudes and behaviors of medical students in Pakistan regarding academic misconduct: A cross-sectional study. BMC Medical Ethics2014;15.	not lived experience	About hypothetical behaviours 
	
Gingerich A. Are PBL tutors identifying struggling students? Med Educ Suppl 2010;44:5.	article type	Congress abstract 	
Ginsburg S, Lingard L. 'Is that normal?' Pre-clerkship students' approaches to professional dilemmas. Med Educ 2011 Apr;45(4):362-71.	not lived experience	students' perceptions based on scenarios	
Ginsburg S, Regehr G, Lingard L. The disavowed curriculum: understanding student's reasoning in professionally challenging situations. J Gen Intern Med 2003 Dec;18(12):1015-22.	not lived experience	students' perceptions based on scenarios 	
Ginsburg S, Regehr G, Lingard L. To be and not to be: the paradox of the emerging professional stance. Med Educ 2003 Apr;37(4):350-7.	not lived experience	students' perceptions based on scenarios	
Ginsburg S, Regehr G, Stern D, Lingard L. The anatomy of the professional lapse: bridging the gap between traditional frameworks and students' perceptions. Acad Med 2002;77:516-522.	population	Not exclusively students behaviours 	
Glick SM. Cheating at medical school. BMJ 2001;322:250-251.
	article type	Letter, no actual behaviours mentioned	
Grimm L, Maxfield C. A proposal to reduce misrepresentation of medical student research activities in ERAS. Acad Med 2014;89:833.	article type	Letter
Grimm and Maxfield - Exclude. It does not talk only about intentional misrepresentations, but also unintentional ones. It is also not empirical.	
Grogan MA. Professionalism in the preclinical years: Medical students' perspectives. US: ProQuest Information & Learning; 2014.	not lived experience	Perspectives of students 	
Hamilton TE. How Issues of Professional Liability Are Taught in U.S. Medical Schools. Academic Medicine 1991;66:39-40.	not lived experience	no actual behaviours	
Helms LB, Helms CM. Forty Years of Litigation Involving Medical Students and Their Education: I. General Educational Issues. Academic Medicine 1991;66:1-7.	article type	Exclude, this articleis about litigations involving medical students and schools and not about student unprofessional behaviour.	
Henning MA, Ram S, Malpas P, Shulruf B, Kelly F, Hawken SJ. Academic dishonesty and ethical reasoning: Pharmacy and medical school students in New Zealand. Medical Teacher 2013;35:e1211-e1217.	population	pharmacy and med students, no distinction can be made 
	
Herzog DB, And O. Substance Use, Eating Behaviors, and Social Impairment of Medical Students. Journal of Medical Education 1987;62:651-657.	definition	No school behaviours	
Hicks, P. J., Cox, S. M., Espey, E. L., Goepfert, A. R., Bienstock, J. L., Erickson, S. S., ... & Peskin, E. (2005). To the point: medical education reviews—dealing with student difficulties in the clinical setting. American journal of obstetrics and gynecology, 193(6), 1915-1922.	article type	opinion 	
Hickson, G. B., Pichert, J. W., Webb, L. E., & Gabbe, S. G. (2007). A complementary approach to promoting professionalism: identifying, measuring, and addressing unprofessional behaviors. Academic Medicine, 82(11), 1040-1048.	population	Descriptions of students and physicians cannot be separated	
Hilton SR, Slotnick HB. Proto-professionalism: How professionalisation occurs across the continuum of medical education. Medical Education 2005;39:58-65.	article type	No research outcomes
	
Hoffman LA, Shew RL, Vu TR, Brokaw JJ, Frankel RM. Is Reflective Ability Associated With Professionalism Lapses During Medical School? Acad Med 2016 Jan 12.	not lived experience	Not about actual unprofessional behaviours	
Holtzman JM, Beck JD, Coggan PG. Geriatrics program for medical students: II. Impact of two educational experiences on student attitudes. Journal of the American Geriatrics Society 1978;26:355-359.	definition	Not about unprofessional behaviours	
Isaacs D. Medical students behaving badly. Journal of Paediatrics and Child Health 2010;46:773.	article type	Letter
No actual behaviours	
Jackson EW, Dawson-Saunders B. History of not completing courses as predictor of academic difficulty among first-year students. J Med Educ 1987;62:880-885.	definition	No unprofessional behaviours but academic difficulty
	
Jamieson J, Mitchell R, Le Fevre J, Perry A. Bullying and harassment of trainees: An unspoken emergency? EMA Emerg Med Australas 2015;27(5):464-7.	article type	Viewpoint
	
Jarmulowicz M. Cheating at medical school. Public declaration of an appropriate punishment is important. BMJ 2001;322:298.	article type	Letter , no actual behaviours	
Jawaid M, Khan MH, Bhutto SN. Social network utilization (Facebook)&e-Professionalism among medical students. Pak J Med Sci 2015;31:209-213.	setting	Behaviours not related to medical school context
	
Jensvold MF, Mackey B, Young-Horvath V. Sexual harassment in medical training. N Engl J Med 1993;329:661-662.	article type	Letter,
No actual behaviours described	
Johnson AC, El Hajj SC, Perret JN, Caffery TS, Jones GN, Musso MW. Smartphones in medicine: emerging practices in an academic medical center. J Med Syst 2015;39:164.	population	Students' behaviours are not reported separately
	
Johnston J, Steele K, McGlade K, Cupples ME. Barriers to professional development in medical students: Revealing the influence of the hidden curriculum. Med Educ Suppl 2010;44:73.	article type	Congress abstract 	
Jones P, Rigby B, Le DC. Professionalism issues identified through workplace based assessment predicts poor performance of medical students in formal written and clinical examinations. Intern Med J  2012;42:30.	not lived experience	No behaviours are mentioned 
	
Kaiser F. Ragging in medical colleges. Rawal Med J 2008;33:106-107.
	article type	View Point, No actual unprof behaviour described
	
Karnieli-Miller O, Vu TR, Holtman MC, Clyman SG, Inui TS. Medical students' professionalism narratives: a window on the informal and hidden curriculum. Acad Med 2010 Jan;85(1):124-33.	population	not about students' unprofessional behaviour	
Kay J, Daniels RS. Shifting psychological patterns of medical students in the 1970s and 1980s. Journal of American College Health 1982;31:133-138.	article type	Viewpoint, No actual unprof behaviour described	
Kellner R, Wiggins RJ, Pathak D. Distress in medical and law students. 
Comprehensive Psychiatry 1986;27:220-223.
	not lived experience	No actual behaviours described	
Kelly M, Oâ€™Flynn S, McLachlan J, Sawdon MA. The Clinical Conscientiousness Index: A valid tool for exploring professionalism in the clinical undergraduate setting. Academic Medicine 2012;87:1218-1224.	not lived experience	No actual behaviours described 	
Kennedy TJT, Regehr G, Baker GR, Lingard LA. Itâ€™s a cultural expectationâ€¦â€™ the pressure on medical trainees to work independently in clinical practice. Medical Education 2009;43:645-653.	definition 	Not about unprofessional behaviours	
Kirsling RA, Kochar MS. Suicide and the stress of residency training: A case report and review of the literature. Psychological Reports 1989;64:951-959.	population	residents	
Kory WP, Crandall LA. Nonmedical drug use patterns among medical students. Int J Addict 1984;19:871-884.	setting	Drug use
Mentions self reported adverse behaviours and impairments	
Krone CR, Rouse SV, Bauer LM. Relationship between perfectionism and academic cheating. Psi Chi Journal of Psychological Research 2012;17:59-67.	population	Not about med students	
Kukolja TS, Taradi M, Knezevic T, Dogas Z. Students come to medical schools prepared to cheat: a multi-campus investigation. J Med Ethics 2010;36:666-670.	setting	self-reported by medical students about (high school education dishonesty)
	
Kumar P, Basu D. Substance abuse by medical students and doctors. J Indian Med Assoc 2000;98:447-452.	article type	Exclude as it is a review	
Lavin B, Pangaro L. Internship Ratings as a Validity Outcome Measure for an Evaluation System To Identify Inadequate Clerkship Performance. Academic Medicine 1998;73:998-1002.	not lived experience	no description of obeserved or admitted behaviour 	
Lazarus LW, Weinberg J. Training in geropsychiatry: Problems and process. The American Journal of Psychiatry 1981;138:1366-1369.	population	residents	
Leblanc T. Early evidence of unprofessional behavior found in medical student records. Virtual Mentor 2007;9:290-294.	article type	Letter, no actual behaviours	
Lee HM. Interns' professional knowledge and professional identity formation in online peer-led dialogue. US: ProQuest Information & Learning; 2014.	population	Not about medical students	
Lipak J, Szombati G, Kleininger O. Preference of visual discrimination factors in childhood. Studia Psychologica 1976;18:292-306.	definition	Not about PB	
London S. Medical students' professional identity and lapses of professionalism in the learning environment. Int J Ostheopath Med 2015;18(1):73-4.	article type	congress abstract	
Lowenfels AB. Unprofessional behavior among medical students. N Engl J Med 2006;354:1851-1853.	article type	Letter, no actual  behaviours	
MacQueen R. Cheating at medical school. Keeping quiet about cheating will not increase public confidence. BMJ 2001;322:297-298.	article type	Letter, No unprof behaviour described 
Reactive op Smith	
Madeeh HA, Rehman A, Butt Z, Awais AM, Shahid A, Abbas KS. Gender Discrimination among Medical Students in Pakistan: A Cross Sectional Survey. Pak J Med Sci 2013;29:449-453.	population	About faculty	
Major A. To bully and be bullied: harassment and mistreatment in medical education. Virtual Mentor 2014;16:155-160.	article type	Editorial, No unprof behaviour described	
McAuliffe WE, Rohman M, Wechsler H. Alcohol, substance use, and other risk-factors of impairment in a sample of physicians-in-training. Advances in Alcohol & Substance Abuse 1984;4:67-87.	definition 	impaired physicians	
McAuliffe WE, Santangelo S, Magnuson E, Sobol A, Rohman M, Weissman J. Risk factors of drug impairment in random samples of physicians and medical students. International Journal of the Addictions 1987;22:825-841.	definition	Not about unprofessional behaviour	
McDonald S. Reply: Bullying at Barts. The Lancet 2012;379:1483.	article type
	Letter, no actual behaviours	
McManus IC, Lissauer T, Williams SE. Detecting cheating in written medical examinations by statistical analysis of similarity of answers: Pilot study. BMJ: British Medical Journal 2005;330:1064-1066.	population	About a computer program	
Meo SA, Usmani AM. Bullying of medical students.  J Coll Physicians Surg Pak 2011;21:579.	article type	Letter, 
No behaviour meniënde	
Miller E, Balmer D, Hermann N, Graham G, Charon R. Sounding narrative medicine: Studying students' professional identity development at Columbia University College of Physicians and Surgeons. Academic Medicine 2014 Feb;89(2):335-42.	not lived experience	focus group discussions about lessons learned in seminars	
Mittal PK, Meena PS, Solanki RK. Problematic use of social networking sites among first year medical students. Indian J Psychiatry 2012;54:S113.	definition	Self-reported use of network sites is related to psychological stress and mental health problems (depression )
Reports no unprofessional behaviours in med school	
Monrouxe LV, Rees CE, Endacott R, Ternan E. 'Even now it makes me angry': health care students' professionalism dilemma narratives. Med Educ 2014;48:502-517.	population	Not about medical students 	
Monrouxe LV, Rees CE, Hu W. Differences in medical students' explicit discourses of professionalism: acting, representing, becoming. Med Educ 2011 Jun;45(6):585-602.	not lived experience	Students' definitions and discourses of professionalism were studied 	
Monrouxe LV, Rees CE, Poole R, Madav U, John D, Oliver R. Healthcare students' narratives of professionalism dilemmas: A multi-centre study of workplace learning. Med Educ Suppl 2011;45:34.	article type	Congress abstract 	
Moore T, Meagher FM. The professional/ethical dilemmas faced by medical students when learning digital rectal examinations. Ir J Med Sci 2014;183(4):S133.	article type	Congress abstract	
Moscarello R, Margittai KJ, Rossi M. Differences in abuse reported by female and male Canadian medical students. CMAJ  1994;150:357-363.	population	Reported mistreatment, not described by medical students	
Mullan CP, Shapiro J, McMahon GT. Interns' experiences of disruptive behavior in an academic medical center. J Grad Med Educ 2013;5:25-30.	population	graduates	
Nicholls GJ. Detecting cheating in written medical examinations: teach, and students may learn something. BMJ 2005;330:1510.	article type	Reactive op Mc Manus, No unprof behaviour described
	
Nishiyama M, Hashimoto M, Tadokoro N et al. Effects of lifestyle-related factors on first- and second-year medical students' fitness to practice. Dokkyo J Med Sci 2013;40:175-184.	definition	
Unhealthy behaviours, not unprofessional behaviours 	
Nora LM, McLaughlin MA, Fosson SE et al. Gender Discrimination and Sexual Harassment in Medical Education: Perspectives Gained by a 14-School Study. Academic Medicine 2002;77:1226-1234.	population	Experienced behaviours, not by med students	
Norman ID, Aikins M, Binka FN. Sexual harassment in public medical schools in Ghana. Ghana Med J 2013;47:128-136.	population	Experienced behaviours, not by med students	
Oancia T, Bohm C, Carr T, Cujec B, Johnson D. The influence of gender and specialty on reporting of abusive and discriminatory behavior by medical students, residents and physician teachers. Medical Education 2000;34:250-256.	population 	selfreport of experienced behaviours, not exclusively by medical students 	
O'Flynn S, Kelly MA, Bennett D. Professionalism and identity formation: Students journeys and emotions. Medical Education 2014 May;48(5):463-5.	article ype	commentary	
Ogoshi K. Academic harassment. Lancet 2001;357:396-397.	population
	Graduate students	
O'Loughlin C. Cheating at medical school. Cheating should be treated like medical error. BMJ 2001;322:297.	article type	Reactive op Smith BMJ 2001, No unprof behaviour described	
Omololu CB. Communication behaviors of undergraduate medical students before and after training. British Journal of Medical Psychology 1984;57:97-100.	not lived experience	Simulated situations 	
O'Neill WM. Patients' complaints: Students must learn communication skills [28]. Br Med J 1993;307:1427.	article type	No behaviours 
letter	
Osman A, Wardle A, Caesar R. Online professionalism and Facebookâ€”Falling through the generation gap. Medical Teacher 2012;34:e549-e556.	population	Students reported unprofessional online behaviour by others. 	
Page M. Medical students accused of cheating. Nat Med 1997;3:1058.	article type	Letter
Behaviours not actually seen	
Park K, Kim MH, Jun HC. Psychiatric variables (alcohol and internet use behavior, anxiety, depression) and academic achievement in premedical students in a Korean university. Eur Child Adolesc Psychiatry 2011;20:S182.	definition	Not about onprofessional behaviours, but unhealthy behaviours	
Parrish DM. Scientific misconduct and findings against graduate and medical students. Sci Eng Ethics 2004;10:483-491.	population 	Graduate students 	
Pascoe H, West J. A clinical approach to the management of unprofessional behaviour in medical students. Med Educ Suppl 2011;45:97-8.	article type	Congress abstract 	
Paton J. Cheating at medical school. Main impact of cheating is on clinical work. BMJ 2001;322:298.	article type	Reactive op Smith BMJ, No unprof behaviour described	
Pope KS. Ethical and malpractice issues in hospital practice. American Psychologist 1990;45:1066-1070.	population	No students	
Rackoff WR. Unprofessional behavior among medical students. N Engl J Med 2006;354:1851-1853.	article type	Letter reacting on Papadakis 2006
No actual behaviours mentioned	
RadovanoviÄ‡ Z, EriÄ‡ L, JevremoviÄ‡ I. The effect of re-testing on the validity of the General Health Questionnaire. Social Psychiatry and Psychiatric Epidemiology 1988;23:36-38.	definition	Psychological impairment , not unprof behaviour in school	
Radstone SJ. Worrying behavior on electives. Practising on the poor? BMJ 2009;338:b519.	article type	Letter, No unprof behaviour described
No behaviours 	
Rai D, Gaete J, Girotra S, Pal HR, Araya R. Substance use among medical students: time to reignite the debate? Natl Med J India 2008;21:75-78.	definition	Unhealthy behaviours, not unprofessional behaviour in school	
Reid A. Identifying medical students at risk of subsequent misconduct. BMJ 2010;340:c2169.	article type	Editorial about Yates' paper, No unprof behaviour described	
Rennie SC, Crosby JR. Students' perceptions of whistle blowing: Implications for self-regulation. A questionnaire and focus group study. Medical Education 2002;36:173-179.	not lived experience 	Hypothetical behaviours . 
	
Rennie SC, Rudland JR. Differences in medical students' attitudes to academic misconduct and reported behavior across the years: A questionnaire study. Journal of Medical Ethics: Journal of the Institute of Medical Ethics 2003;29:97-102.	not lived experience 	Hypothetical behaviours	
Rennie, S.C. & Crosby, J.R. 2001. Are "tomorrow's doctors" honest? Questionnaire study exploring medical students' attitudes and reported behavior on academic misconduct. BMJ, 2001;322, (7281) 274-275	not lived experience	Hypothetical behaviours	
Richardson DA, Becker M, Frank RR, Sokol RJ. Assessing Medical Students' Perceptions of Mistreatment in Their Second and Third Years. Academic Medicine 1997;72:728-730.	population 	Students experience mistreatment by faculty 
	
Richardson R. Cheating in medical school. Medical students cheated in the past too. BMJ 1995;311:194.	article type	Letter
Reaction op Leavitt, No unprof behaviour described	
Robb N. Racism can rear its ugly head at medical school, study finds. CMAJ 1998;159:66-67.	article type	no scientific article	
Robinson D, Garner J, O'Sullivan H. Safety first! Professional behaviour of medical students online. Med Educ Suppl 2010;44:76.	article type	Congress abstract 	
Rockey DC. Unprofessional behavior among medical students. N Engl J Med 2006;354:1851-1853.	article type	Letter reacting on Papadakis 2006
No actual behaviours	
Rubin P. When medical students go off the rails: Student support is essential, but so is protecting the public. BMJ: British Medical Journal 2002;325:556-557.	article type	Letter, No unprof behaviour described	
Salam A, Song CO, Mazlan NF, Hassin H, Lee LS, Abdullah MH. Professionalism of future medical professionals in Universiti Kebangsaan Malaysia (UKM) Medical Centre. Int Med J 2012 Sep;19(3):224-8.	not lived experience	Questionnaires to students about elements of professionalism	
Santen SA, Petrusa E, Gruppen LD. The relationship between promotions committeesâ€™ identification of problem medical students and subsequent state medical board actions. Advances in Health Sciences Education 2014.	not lived experience 	“behavioural issues” no further explication , No unprof behaviour described	
Sareen J, Spiwak R, Isaak C, Mullins M, Barakat S. Medical students and postgraduate Residents observations of unprofessional online postings on social networking sites. Med Educ Suppl 2010;44:14.	article type	Congress abstract 	
Scheiber SC, Henderson PB. The impaired trainee. In: Cohen RL, Dulcan MK, Cohen RL, Dulcan MK, eds. Basic handbook of training in child and adolescent psychiatry. Springfield, IL, England: Charles C Thomas, Publisher; 1987;247-273.	population 	residents	
Scott J, O'Dea A, O'Connor P, Byrne D. An analysis of lapses in professionalism as experienced by interns in clinical practice. Ir J Med Sci 2014;183(4):S139.	article type	Congress abstract	
Seshadri S. Substance abuse among medical students and doctors: a call for action. Natl Med J India 2008;21:57-59.	article type	Editorial, No unprof behaviour described	
Shea JA, O'Grady E, Morrison G, Wagner BR, Morris JB. Medical student performance evaluations in 2005: An improvement over the former dean's letter? Academic Medicine 2008;83:284-291.	population 	About deans letters
Not behaviours of students mentioned	
Shyangwa PM, Joshi D, Lal R. Alcohols and other substance use/abuse among junior doctors and medical students in a teaching institute. JNMA J Nepal Med Assoc 2007;46:126-129.	definition	Unhealthy behaviours, not unprofessional behaviours in school	
Siden H, Ticho B, Kopnick M. Malpractice concerns enter the medical school classroom. N Engl J Med 1986;314:522-523.	not lived experience	No behaviours described	
Sierles FS, Brodkey AC, Cleary LM et al. Medical students' exposure to and attitudes about drug company interactions: a national survey. JAMA 2005;294:1034-1042.	not lived experience 	About attitudes
	
Smith R. Cheating at medical school. BMJ 2000;321:398.	article type
	Smith, 2000; editorial, No unprof behaviour described	
Smith RC. Teaching interviewing skills to medical students: The issue of 'countertransference.'. Journal of Medical Education 1984;59:582-588.	definition	Not about unprofessional behviour	
Spencer SA. Cheating at medical school. Anonymous letter should have been consigned to the bin. BMJ 2001;322:296.	article type	Letter, No unprof behaviour described	
Spiwak R, Mullins M, Isaak C, Barakat S, Chateau D, Sareen J. Medical students' and postgraduate residents' observations of professionalism. Education for Health: Change in Learning & Practice 2014;27:193-199.	not lived experience	Hypothetical behaviours.	
Stern DT, Frohna AZ, Gruppen LD. The prediction of professional behavior. Medical Education 2005;39:75-82.	not lived experience	Describes predictors for unprofessional behaviour	
Stimmel B, Yens D. Cheating by medical students on examinations. Am J Med 1982;73:160-164.	population	Deans attitudes towards cheating	
Stone JP, Charette JH, McPhalen DF, Temple-Oberle C. Under the knife: Medical student perceptions of intimidation and mistreatment. J Surg Educ 2015;72(4):749-53.	population	Not about unprofessional behaviours of students	
Stoner EN, Schupansky SP. Disciplinary and Academic Decisions Pertaining to Students: A Review of the 1997 Judicial Decisions. Journal of College and University Law 1998;25:293-312.	population 	residents and students, not described separately	
Tan E. Cheating at medical school. Public horsewhipping is not the answer. BMJ 2001;322:296-297.	article type	Letter, No unprof behaviour described	
Thomas K, Duffy H, Calvert M. Can we predict students that show signs of unprofessional behaviour? The use of yellow cards. Med Educ Suppl 2010;44:114.	article type	Congress abstract 	
Thompson JA. Cheating in medical school. Lying statement reinforces in appropriate cultural stereotype. BMJ 1995;311:194.	article type	Letter 
No unprof behaviour described	
Tolkin L, Glick S. [Ethical behavioral standards of medical students on examinations and studies]. Harefuah 2007;146:429-34, 502.	not lived experience 	Only perceptions and attitudes	
Van der Veer T, Frings-Dresen MHW, Sluiter JK. 
Health behaviors, care needs and attitudes towards self-prescription: A cross-sectional survey among Dutch medical students. PLoS ONE 2011;6.	definition 	About unhealthy behaviour, not about unprofessional behaviour	
Vengoechea J, Ruiz Ãl, Moreno S. EstrÃ©s y conductas antidisciplinarias en estudiantes de una facultad de medicina de BogotÃ¡. = Stress and misconduct in students of a medical school in BogotÃ¡. Revista Colombiana de PsiquiatrÃ­a 2006;35:340-351.	definition 	About stress, not about unprofessional behaviour 	
Vive J. Cheating at medical school. Examination committee's decision tarnishes reputations. BMJ 2001;322:297.	article type	Letter, no actual behaviours described	
Wagner RF. Medical student academic misconduct: Implications of recent case law and possible institutional responses. Academic Medicine 1993;68:887-889.	article type	no actual behaviours described	
Watts GT. Cheating in medical school. Poor teaching may be responsible. BMJ 1995;311:193-194.	article type	Letter, no actual behaviours described
	
Wear D, Aultman J. Sexual harassment in academic medicine: Persistence, non-reporting, and institutional response. Medical Education Online 2005;10:1-11	population 	Perceptions of medical students about faculty bahaviour	
Westermeyer J. Substance use rates among medical students and resident physicians. JAMA 1991;265:2110-2111.	article type	Editorial, no actual behaviours described	
Westlake W. Cheating at medical school. Committee should be commended for showing compassion. BMJ 2001;322:297.	article type	Letter, no actual behaviours described	
White GE. Sexual harassment during medical training: The perceptions of medical students at a university medical school in Australia. Medical Education 2000;34:980-986.	population 	Perceptions of medical students about faculty behaviour	
White J, Kirwan P, Lai K, Walton J, Ross S. 'Have you seen what is on Facebook?' the use of social networking software by healthcare professions students. BMJ Open 2013;3.	population 	Not exclusively medical students	
Whittle SR, Murdoch-Eaton DG. Learning about plagiarism using Turnitin detection software. Med Educ 2008;42:528.	not lived experience	Not about students' actual unprofessional behaviour	
Wright N. Bullying at Barts. The Lancet 2012;379:1483.	not lived experience	No actual behaviours described
	
Yates J, James D. Risk factors at medical school for subsequent professional misconduct: Multicentre retrospective case-control study. BMJ: British Medical Journal 2010;340.	population 	Physicians	
