# Supplementary material for: Descriptors for unprofessional behaviours of medical students: a systematic review and categorisation
Source: BMC Med Educ. 2017 Sep 15;17:164. doi: 10.1186/s12909-017-0997-x (PMC5603020; doi:10.1186/s12909-017-0997-x)
Supplement: Supplementary file 3 — Included 46 studies. (RTF 530 kb) [file 12909_2017_997_MOESM3_ESM.rtf]

Additional file 3_ Included 46 articles 


Study 	Design	Methods
	Witnessed/admitted unprofessional behaviours of medical students	Quality	
Aderounmu et al.,201150
		
			in
	Cross sectional survey

	Questionnaires to students of four medical colleges in Nigeria, 382 students responded, no response rate given	Parent seeks exam materials 	
Let someone else sit for your University qualifying Exams	
Writing a full exam for another person	
Copying another student's work/ assignment                      
Allowing others to copy your work                               
Copying answers in a university exam before sitting for it
Copying others laboratory results without performing any test	
Writing clinical exam “normal" when you didn't perform 	
Smuggling materials to cheat into an exam hall	3	
Ainsworth & Szauter, 200615 
	Case study using qualitative analysis of student records	Unprofessional behaviours of 90 students described in 103 “Early Concern Notes” at one US medical school were compared with behaviours of 516 disciplined physicians 	Failure to fulfill responsibilities reliably
Misrepresents or falsifies actions/information
Inadequate personal commitment to patients
Resistant or defensive in accepting criticism 
Inadequate rapport with patients/families
Does not function /interact appropriately within groups
Fails to accept responsibility for actions
Unaware of limits
Insensitive to needs, feelings of others
Fails to respect patient confidentiality
Accepts/seeks minimally acceptable level of performance
Uses disrespectful language
Abuses student privileges
Impairment 
Arrogant or abusive during stress
Fails to maintain professional appearance and attire	5	
Anderson & Obershain, 199447
	Cross sectional survey	Questionnaires to 341 faculty and 291 students at one US medical school, response rate 60%	Copying from another student during an end-of-block examination
Permitting another student to copy from you during an end-of-block examination
Observing a student copying from another student during an examination and doing nothing with the information
Copying from a 'crib sheet” or another student during a closed book examination
Reporting a lab test or X-ray as “normal” during rounds when in actual  ordered or knew it had not been
Reporting a pelvic examination as “normal” when it had been inadvertently omitted)from the physical examination
Taking an examination for another student
“Previewing” a stolen copy of Part1 National Boards the night before the exam
Writing up a laboratory exercise in biochemistry without having done the work
Working with a group of students on an assignment designed to be  carried out individually
Delaying taking an examination using a false excuse
Looking at another students' examination and keeping your answer if both answers are the same
Removing an assigned reference from the reserved shelf in the library, thereby preventing other students from gaining access to the information
Changing a response after a quiz was graded and returned, then reporting that there had been a mistake and requesting credit from the altered response
Plagiarizing a report for a biochemistry paper from a friend's paper from a prior year	5	
Babu et al., 201142
	
	

	Cross sectional survey	Questionnaire to 1268 undergraduate students from four private medical schools in India. 166 responses were analysed.
Respons rate 13%	Giving proxy for attendance
Copying blindly from somebody's record
Copying during exams
Copying from unauthorised study material during their exams
Trying to get an exam question paper before the commencement of the exam
Influencing the teacher to get more marks
Getting technical help during practical exam
Getting help in knowing the findings of an exam case
Mentioning a system as “within normal limits” without examining
Forging teachers' signature in their record or log books	4	
Baldwin & And, 199653 
	Cross sectional survey	Questionnaire to 3975 second year medical students at 31 US schools,
2459 responses were analyzed, respons rate 62%	Copying answers on a test
Getting a copy of a test prior to an exam
Getting information about a test from others prior to an exam
Exchanging answers during an exam
Turning in an written assignment prepared by someone else and calling it his or her own
Moving labels or altering slides during an exam
Using a “cheat sheet” during an exam
Taking an exam for someone else 
Altering his or her grades in the official record 	5	
Barlow, 201563	Cross sectional survey	Online survey to all medical students in 20 Australian medicals schools . Of 16 993 eligible students, 880 completed the survey	Online posting of patient identifying material 
Discussing a clinical site in a negative light
Discussing university in a negative light
Discussing another health care worker in a negative light	4	
Bazoukis & Dimoliatis, 201149	Cross sectional survey	Triangulation of four cross sectional surveys using questionnaires about self-reported cheating of students in 7 medical schools in Greece	Cheating in exams
Passing an exam by using help from acquaintances 

	4	
Ben-Yakov , 201566	Cross sectional survey 	530 responses of senior medical students of a Canadian medical school 
Respons rate 49,1%	Use Facebook to research patients
Use Google to research patients	4	
Bilic-Zulle et al., 200559 
	Comparative case series	Essays of 198 medical students, based on complex/less complex sources and electronic/printed sources, were examined using plagiarism software in one medical school in Croatia.	Plagiarism	5	
Burack et al., 19999 
	Multi method study, including qualitative analysis of action-based observations	Multi-method study (observation, thinking aloud task, interviews, patient chart review) of 4 ward teams existing of 1 attending physician, 1 senior resident, 2interns and max 3 students, in one university affiliated hospital in the US.	Showing outright hostility, malice or rudeness
Reluctance in pursuing clinically appropriate diagnostic and therapeutic steps, including avoiding admission, pressing for premature discharge, or otherwise cutting corners
Referring to patients in disparaging or a derogatory ways, or otherwise showing disrespect	5	
Chretien et al., 200962 
	Cross sectional survey	Survey under deans of student affairs in 130 US medical colleges, 
78 completed questionnaires were analysed	On line posting sexual-relational content, i.e. posting sexually suggestive/ explicit content or posting sexually provocative photographs of students, requesting inappropriate friendships with patients on Facebook, sexually suggestive comments.
On line posting negative content related to experiences in medical school, i.e. using profanity or other disparaging or discriminatory language in reference to specific faculty, courses or rotations, classmates, or medical school.
On line posting content like comments, photos and videos suggesting intoxication or illicit substance use. 
On line posting content which posed threats to patient confidentiality, i.e. detailed references to patients putting patient privacy at risk 	5	
Coverdale & Hanning, 200051
	Cross sectional survey 	137 medical students completed a questionnaire addressing their own cheating behaviours. 
Overall response rate 54%.	Altering or manipulating data
Falsifying references or a biography
Reporting an aspect of a physical examination as `normal' when it was inadvertently omitted from the examination
Copying a report for a preclinical or clinical paper from a friend's paper from a prior year
Copying from a neighbour during an examination without the person realizing
Permitting another student to copy from another student during an examination
Removing an assigned reference from a shelf in the library and thereby preventing others from,gaining access to the information in it
Reporting a lab test or X-ray as `normal' during ward rounds when in actual fact there had been no attempt to obtain the information
Taking unauthorised material into an examination
A student taking an examination for someone else or having someone else taking an examination
for him or her	5	
Dans, 199652	Comparative cross sectional survey	Questionnaires to 358 students of one US medical school, at school entry and in fourth year of medical school	Copying from someone else's paper
Using crib notes
Falsifying lab data
Reuse old examinations
Cheating in clinical examinations
Recording tasks that were not performed
Reporting findings that were elicited by others
Lying about having ordered tests	4	
Dyrbye, 201044 


	Cross sectional survey	Questionnaire sent to all medical students attending 7 US medical schools. Overall response rate 61%.
Outcome: self-reported cheating/dishonest behaviours.	Copying from a “crib-sheet” or another student during a “closed-book” examination
Took credit for another person's work
Permitted another student to copy from you during a closed book examination
Said you ordered a test when you actually had not
Reported a laboratory test or x-ray as pending when not sure it was ordered or knew it had not been
Reported result as normal when you knew it had been inadvertently omitted from the physical examination
Signed an attendance sheet for a student who was not present
Endorsed more than one unprofessional behaviours
Acceptance of gifts	5	
Friedman et al, 197865

	Qualitative study based on student observations	Observation of 6 sex education seminars in which 70 students participated	Negative responses in a sex education seminar 
Whispering animatedly about material that was obviously not of general educational value 
Subgroup formation 
Belligerence
Withdrawal
Sleeping in class 
Verbally expressed hostility , eg. posing provocative questions in a challenging manner 
Outright angry disruptive opposition to learning materials	2	
Garner & O'Sullivan, 201064 
	Cross sectional survey	Questionnaires to students from year 1-3 in one medical school in the UK. 56 completed questionnaires, response rate 31 %	Unprofessional behaviours on Facebook:
-	excessive drinking, 
-	various stages of undress and 
-	the discussion of clinical experiences with patients	4	
Hafeez et al., 201343	Cross sectional survey	Students from three medical colleges in Pakistan. 274 completed questionnaires, overall respons rate 33%	Cheating during exams
Using mobile phone to exchange answers during an exam
Trying to find out about test questions in advance 
Marking answers on the question paper during the OSCE/OSPE
Asking the teacher for answers during OSCE
Telling friends the questions which were asked in first shift in the OSCE
Copying assignments/presentations from seniors/class mates
Marking proxy for your friends
Asking friend to mark attendance
Forging teacher's signature
Pay someone to pass an exam
Writing fake histories for assignments
Writing fake examination findings without performing it	5	
Hauer et al.,200730
	Qualitative study using interviews (grounded theory)	Interviews with individuals responsible for remediation from different public and private schools in the US. Data from 33 interviews were analysed until saturation of data was obtained.	Detachment
Emotional distance
Poor verbal communication
Poor nonverbal communication
Fail to elicit the patient's perspective
Fail to determine the impact of psychosocial factors on the patient
Treat simulation patients as symptoms and diagnoses rather than as people with feelings and concerns 
Poor insight
Lack of empathy
Resistance to the examination process 
Dressing too casually during examination, 
Speaking too casually in examination
Arriving late for exam
Chewing gum during exam
Denying own performance
Blaming external factors rather than skill deficiencies for bad exam results 	5	
Heiman et al., 201457 
	Cross sectional survey	Third year student of one US medical school. 123 completed questionnaires, respons rate 75%	Copying and pasting elements of another provider's notes in the electronic health record documentation (EHRD)
Copying  elements of my own previous notes 
Copying elements of residents' notes 
Copying elements of attendings' notes 
copying elements of other students' notes 
using auto-inserted data for vital signs 
using auto-inserted data for lab results 
using auto-inserted data for the medication list 
using templates for the entire note 
using templates for the physical or mental status exam 
using auto-inserted data for the problem list 
Documenting while signed in under an attending's name 
Documenting while signed in under a resident's name	5	
Hejri et al., 201339 
	Cross sectional survey	Questionnaires to clerks and interns of one medical school in Iran.,
124 were analysed, response rate 86%

	Cheating in examinations 
Helping others to cheat in examinations
Gaining illegal access to examination questions
Impersonating an absent person in a class
Escaping teamwork 
Legitimising absences by falsified testimony
Legitimising absences by using bribes 
Data fabrication 
Data falsification 
Plagiarism 
Forging signatures 
Writing thesis on behalf of others 
Fabricating the whole or part of a patient's history
Reporting abnormal physical examination findings as normal
Using other people's medical stamps 
Buying hospital shifts 
Selling hospital shifts 	5	
Hendelman &Byszewski 201438
	Cross sectional survey	Questionnaires to students of one medical school in Canada. 
255 questionnaires were analyzed. 45%Respons rate 	Arrogance
Impairment
Cultural and religious insensitivity
Breach of confidentiality
Lack of conscientiousness
Bias and sexual harassment
Misrepresentation
Collaboration with industry
Acceptance of gifts
Compromising ethical principles	5	
Howe et al., 201041 
	Mixed methods study including qualitative analysis of student records	Mixed methods utilising exam board and administrative data of one UK medical school for statistical and descriptive analysis of unprofessional behaviour of 118 students 	Plagiarism
Collusion
Unexplained/unauthorised absence
Lack of meeting responsibilities
Falsification of signatures
Significant misconduct 	3	
Hrabak et al., 200445
	Cross sectional survey	Questionnaire to year 2-6 medical students of a medical school in Croatia. 
827 were analysed, respons rate 70%	Submitting another student's work under one's name
Paying an examiner to pass an examination
Using private connections to arrange passing an examination
Forging a teacher's signature in a matriculation book
Finding out about test questions in advance
Using a crib sheet during an examination
Using a mobile phone to exchange answers during an examination
Copying answer from a colleague during examination
Arranging with administrative personnel to be assigned to a lenient examiner
Altering a class attendance list
Asking a colleague to sign you in on a class attendance list
Signing in an absent colleague	5	
Hunt et al., 198928 
	Cross sectional survey	Questionnaires to residents and teachers of several medical disciplines of one medical school in the US about observed behaviour of medical students in their clinical rotations . 466 responses were analyzed, respons rate 79%.	Bright with poor interpersonal skills
Excessively shy, non-assertive
Poor integration skills
Cannot be trusted
Over-eager
Hostile
Cannot focus on what is important 
Disorganised 
Rude
Disinterested 
Too informal 
Avoids work
Avoids patient contact
Does not show up
Challenges everything
'All thumbs' (clumsy)
'Con artist '- (manipulative behaviour) 
A poor fund of knowledge
Does not measure up intellectually	5	
Kapoor, 201668	Cross sectional survey	Questionnaires to 400 students at 1 medical school in India	Bullying
Physical
Verbal
Victimization	4	
Kulac et al., 201331 	Cross sectional survey	Survey of 215 year 3 and 4 students of one Turkish medical school. 215 responses were analysed, respons rate 68%. 	Making fun of patients, peers, or physicians 
Being introduced as “doctor” to patients 
Reporting an impaired colleague to faculty before approaching the individual 
Poor condition of white coats 
Taking food meant for patients 
Discussing patients in public spaces 
Making derogatory comments about patients 
Discuss with patients information beyond your level of knowledge 
Late to rounds 
Absent from mandatory lectures 
Wear white coats/scrubs in a nonclinical environment (e.g., the cafeteria) 
Wear white coats/scrubs out of the hospital (e.g., the hospital courtyard) Untidy dress 
Not correcting someone who mistakes you for a physician 
Being introduced as “student doctor” to patients 
Eating or drinking in patient corridors 
Taking food from lectures you are not attending 
Having personal conversations in patient corridors	5	
Monrouxe, 201256	Qualitative study with narrative interviewing of individuals or groups	Qualitative analysis of 200 narratives of a convenience sample of 833 students from 2 UK and 1 Australian  medical school.
	No consent for clinical examination of a patient 
Placing own learning above patient safety 
Judgmentally talking about patients 
Misrepresentation
Acting beyond their own levels of competence 
Poor hand-washing practice	5	
Mukhtar et al., 201067	Cross sectional survey	Survey of first and fourth year students of one medical school in India.  106 completed questionnaires, overall response rate 53%. Students report about abuse of peer students.	Physical abuse
Written abuse
Ignoring and excluding a peer student 
Behavioural abuse
Verbal abuse	5	
Papadakis et al., 199934 
	Case study using qualitative analysis of student records	29 reports of 24 students presented to the dean's office were analysed	Lack of initiative
Unmet professional responsibility
Poor relationship with team
Poor rapport with patients and families
Arrogant
Falsifies information
Resistant to change
Unaware of inadequacies
Resistant to criticism
Avoided patients
Disruptive with team
Lack of interest
Inappropriate dress
Lack of timeliness
Argumentative
Lack of effort towards self-improvement
English language difficulties	3	
Papadakis et al., 200437 

	Comparative case control study	Behaviours of 68 graduates of one US medical school , who were disciplined by a state medical board, were compared with 196 non-disciplined graduates to find predicting variables of professionalism issues	Immature 
Resistant to accepting feedback
Needs continuous reminders to fulfill ward responsibilities
Unnecessary interruption in class
Inappropriate behaviour in small groups with peers and with faculty
Cannot work with peers	5	
Papadakis et al., 200535 
	Comparative case control study	235 graduates of three US medical schools who were disciplined by a state medical board were compared with 469 non disciplined graduates, to find predictor variables of professionalism issues
	Irresponsibility
Diminished capacity for self-improvement  
Immaturity
Poor initiative 
Impaired relationships with students, residents or faculty
Impaired relations with nurses
Impaired relationships with patients and families
Unprofessional  behaviour associated with anxiety, insecurity or nervousness	5	
Parker et al., 20085	Case study using qualitative analysis of student records	Evaluation of a teaching program at one medical school in Australia, including the description of students'unprofessional behaviours from 291 “needs assistance reports”	Unsatisfactory responsibility/reliability
Unsatisfactory participation 
Unsatisfactory respect
Unsatisfactory relating to others
Unsatisfactory self-appraisal
Unsatisfactory honesty/integrity
Unsatisfactory compassion
Unsatisfactory doctor/patient relationship
Discrimination	3	
Phelan & And, 199314
		Q	IN	Case study using qualitative analysis of student records	Evaluation of a PB assessment system at one medical school in the US. Analysis of 32 assessment forms indicating non-cognitive behaviours that caused concern 	Inappropriate behaviour in lecture, 
Negative attitude, 
Seems to feel put upon when asked to do authority, 
Students do not want to work with him, 
Manipulative, aggressive, and badgering of faculty, 
Doesn't respond to written requests to discuss low grades, 
Non-participating, seems withdrawn.	3	
Reddy et al., 200732 
	Comparative cross sectional survey	Pre and post-survey of medical students' perception and observation of, and participation in unprofessional behaviours at one US medical school.
61 participants, response rate 62% 	Arriving late to rounds 
Absent from mandatory lectures
Women's dress
Men's dress
Making fun of patients, peers or physicians
Not correcting someone who mistakes you for a physician
Being introduced as doctor or student-doctor to patients
Reporting an impaired colleague to faculty before approaching the individual
Poor condition of white coats
Taking food from lectures you are not attending
Discussing patients in public spaces
Making derogatory comments about patients
Inebriation at school events
Discuss with patients information beyond your level of knowledge
Consent a patient for minor procedures without supervision
Perform procedures beyond your level of skill on patient
No feedback to residents or faculty regarding their unprofessional behaviour
Unclear expectations or insufficient feedback by faculty or residents	5	
Rees, 201355	Cross sectional survey	Quantitative thematic and discourse analysis of 680 narratives about professionalism dilemmas  written by students who responded to an  online questionnaires (n=2327) sent to all students of 29 UK medical schools.
Respons rate not given.	Communication violation to patients
Communication violation about patients
Breaching patients' confidentiality
Putting own learning needs ahead of patient care,  and thereby causing the patient discomfort
Acting beyond level of competence
Participating in examinations or procedures with no or invalid patient consent	5	
Shukr & Roff, 201446


	Cross sectional survey	Student survey in two Pakistani medical colleges. 480 completed questionnaires with self-reported unprofessional behaviour of medical students. Respons rate 92%.	Take the work or idea from a fellow student and passing it off as one's own without acknowledging it or purchasing work from a supplier
Getting or giving help for coursework, against a teachers rule (e.g. lending work to another student to look at
Claiming collaborative work as one's individual effort 
Paying a fellow student, or being paid by a fellow student,for completion of coursework
Resubmitting work previously submitted for a separate assignment or earlier work
Intentionally paraphrasing text in an assignment, or copying text directly, without acknowledging the source
Failing to correctly acknowledge a source (e.g., copying the text directly but only including the source in reference list)
Citing sources that have not in fact been read in full 
Altering or manipulating data (e.g., adjusting the data to obtain a significant result)
Removing an assigned reference from the shelf in the library in order to prevent other students from gaining access to the information in it
Deliberately damaging another students' work 
Attempting to use personal relationships, bribes, or threats to gain academic advantage
Copying answers from a neighbor or enabling a neighbor to copy your answers during an exam
Exchanging answers using mobile phones during an exam 
Receiving information about the paper from a student who have already sat in the exam, or providing information about a paper to students who have yet to sit in it
Persuading faculty members into providing copies of paper prior to exam through bribery, force or threat
Taking unauthorised materials (e.g., crib sheets, ''Bootee'') into an exam
Sitting an examination for someone else, or someone else s an examination for you
Inventing unrelated or irrelevant circumstances to delay sitting in an exam
Arranging to pass an exam using private connections, or bribery
Signing attendance sheet for absent friends, or asking classmates to sign attendance sheets for you in labs or lectures
Missing lectures frequently  
Intentionally falsifying the test results or treatment records in order to disguise mistakes 
Failing to follow proper infection control procedures  
Examining patients without knowledge or consent of supervising clinician
Forging a health care worker's signature on a piece of work, patient chart, grade sheet, or attendance sheet
Falsifying references or grades on curriculum vitae 
Altering grades in official record 
Sexually harassing a university employee or fellow student 
Threatening or verbally abusing a university employee or fellow student
Physically assaulting a university employee or fellow student 
Engaging in substance abuse (e.g., drugs) 
Providing illegal drugs to the students 	5	
Sierles & And, 198054 
	Cross sectional survey	Student survey at two US medical schools. 482 completed questionnaires, respons rate 95%	Cheating in exams
Falsifying information about a patient from a laboratory examination, history or physical examination
Reporting a finding on a patient as normal without obtanining the information	5	
Silva-Villarreal et al., 201361
	Cross sectional survey 	Student survey with questionnaires in one medical school in Panama. 472 participants, respons rate not given	Bullying: 
Stealing or breaking things
Threatening others
Spreading rumours
Profanity
Exclusion
Insulting	5	
Simpson, 197758
	Qualitative study using interviews	Interviews with doctors, medical students and staff attorneys at four US teaching hospitals, by a law-scholar	Misrepresentation	3	
Subba et al., 201360
	Cross sectional survey	Student survey about mobile phone usage at one medical school in India, 336 completed questionnaires, response rate not given	Use of phones in restricted areas

	4	
Taradi & Taradi, 201248 
	Cross sectional survey	Student survey among students of four Croatian medical schools, 662 completed questionnaires, respons rate 62%	Turning in work done by someone else 
Getting exam questions from someone who already has taken the test
Helping someone else cheat on a test 
Copying from another student during a test or exam without his/her knowledge
Copying from another student during a test or exam with his/her knowledge
Copying text without appropriate attribution 
Using unpermitted crib notes during a test 
Taking a test or a part of a test for someone else
Allowed someone else to copy from your test 
Using false excuse to obtain extension on due date	5	
Teherani et al., 200536 
	Case study using qualitative analysis of student records	Retrospective qualitative analysis of 68 student cases (disciplined) and 196 matched cases (nondisciplined). 	Poor reliability and responsibility 
Lack of self-improvement and adaptability 
Poor initiative and motivation 
Immaturity 
Poor relationships with students, faculty, staff 
Poor relationships with patients and patient families 
Does not uphold medical school honor code 
Apple polisher, show-off, needs to be center of attention 
Anxious, insecure	5	
Teherani et al., 200933 
	Qualitative study using interviews	Interviews with 33 faculty from 33 different US medical schools, responsible for remediation were analyzed by using a validated framework.	Inability to accept and incorporate feedback
Negative attitude
Arrogant
Overconfident
Poor character
Brusque, hostile or argumentative
Falsifies
Undesirable as a physician
Fails to establish rapport
Displays inappropriate interpersonal skills
Not respectful
Insensitive to patient needs
Late or absent for assigned activities
Unreliable
Lacks motivation
Oversensitive	5	
Vengoechea et al., 200840
	Cross sectional survey	Survey in one medical school in Colombia to find perceptions and actual unprofessional behaviours. Questionnaires of 433 medical students were analyzed, overall respons rate 80%	Copying from another in an exam 
Copying literally from published books or articles 
Lending work to another so he/she may copy 
Using downloaded material without reference 
Writing the heart rate in a medical chart without taking it 
Leaving the hospital during a shift 
Presenting work with the name of someone who did not participate in it
Paying someone to do a shift for you 
Obtaining a copy of a test before presenting it 
Asking someone to include you in the assistance list 
Answering 'negative' if asked about patient past history you did not obtain
Paying someone to change a grade	4	
Yates, 201427 
	Case study using qualitative analysis of student records	Case study of students records of one UK medical school, 189 forms of 143 students were analyzed	Inappropriate comments made to a patient in front of others
Inappropriate advice to a patient
Giving other students inappropriate advice about clinical care
Illegible writing
Failing to listen to patients' opinion
Failing to contribute to patient care
Absence from teaching with notice or prior permission
Failure to follow the timetable and/or get assignments signed off
General lack of commitment to teaching & learning activities and/or tutor meetings
Failure to engage with research project, poor note-keeping and general
disorganisation
Ignoring emails or other contacts from teaching or administrative staff
Disruptive behaviour in group teaching sessions
Dismissive or arrogant behaviour to other individuals during teaching
Rudeness to colleague in presence of simulated patient
Making a patient feel uncomfortable during examination
Inconveniencing patients by not attending and not appreciating the  problems caused
Not respecting professional boundaries (deciding to visit a patient at home)
Abrupt and non-empathetic manner with patients
Rude or aggressive to fellow students or to staff, with confrontational,
intimidating or arrogant behaviour
Making fun of others inappropriately
Using offensive language during teaching sessions
Lack of engagement with clinical teams, disrespect, lack of insight into
behaviour
Poor body language, inattention, disinterest and casual behaviour
Plagiarism or fabrication in written work
Failing to obey rules & regulations, particularly in Halls of Residence
Giving false identification when challenged
Drunk & disorderly behaviour in Halls, noise disturbance
Asking another student to sign them in for teaching, or signing another in
themselves
Arrest or criminal offence
Writing rude/inappropriate comments on exam script
Work or attendance affected by health disorders such as depression
Student failing to appreciate the effects of poor health on performance and seek support	4	
Ziring, 201529	Cross sectional survey 	Mixed method analysis of 93 telephone or email interviews with  key administrators of  153 eligible US and Canadian medical schools	Lapses in responsibility: 
Missed deadlines
Unexcused absences
Tardiness
Lapses in relationships
Disrespectful communication by e-mail or in person
Inappropriate use of social media
Poor availability
Lapses related to diminished capacity for self-improvement
Lack of self-awareness
Lack of awareness of one's limitations
Lack of initiative
Being defensive to feedback
Cheating in exams
Committing a felony
Falsifying patient information
Falsifying resident application information
Forging prescriptions
	5	
