# Supplementary material for: Descriptors for unprofessional behaviours of medical students: a systematic review and categorisation
Source: BMC Med Educ. 2017 Sep 15;17:164. doi: 10.1186/s12909-017-0997-x (PMC5603020; doi:10.1186/s12909-017-0997-x)
Supplement: Supplementary file 4 — List of themes, descriptors and behaviours. (DOCX 40 kb) [file 12909_2017_997_MOESM4_ESM.docx]

| **Themes** | **Descriptors** | **Behaviors** |
| --- | --- | --- |
| Failure to engage | Late or absent for assigned activities | Lack of timeliness[28-30,37]  Unexplained/unauthorised absence[26,27,29-32,42,43] |
|  | Not meeting deadlines | Failure to follow the timetable and/or get assignments signed off[26,32] |
|  | Poor initiative | Lack of initiative[32,37,38,70]  Excessively shy, non-assertive [27,28,66]  Avoids patient contact[26,27,37]  Inattention, non-participating[26,33,36,66]  Disinterested[27,37]  Lacks motivation[31]  Negative attitude[31,36]  General lack of commitment to teaching & learning activities and/or tutor meetings[26]  Failure to engage with research project[26]  Lack of engagement with clinical teams[26]  Casual behaviour[26,27] |
|  | General disorganization | General disorganisation[26,27]  Poor note-keeping[26]  Illegible writing[26] |
|  | Cutting corners | Poor reliability and responsibility[25,31,33,34,37,38,42,70]  Inadequate personal commitment to patients[25]  Accepts/seeks minimally acceptable level of performance[25]  Reluctance in pursuing clinically appropriate diagnostic and therapeutic steps, including avoiding admission, pressing for premature discharge, or otherwise cutting corners[39]  Lack of conscientiousness[35]  Avoids work[27,32]  Leaving the hospital during a shift[41] |
|  | Poor teamwork | Does not function /interact appropriately within groups[25]  Escaping teamwork[40]  Cannot work with peers[34]  Disruptive with team[37]  Inappropriate behaviour in small groups with peers and with faculty[34]  Gives no feedback to others[30]  Claiming collaborative work as one’s individual effort[43,46] |
|  | Language difficulties | English language difficulties[37] |
| Dishonest behaviours | Cheating in exams | Cheating in exams[32,40,45,50,53,55]  Gaining illegal access to examination questions[40,43-48,51,53,54]  Paying someone to change a grade[41,45,48]  Let someone else sit for your exams or taking a test or a part of a test for someone else[46,47,51,52,54]  Observing a student copying from another student during an examination and doing nothing with the information[46]  Changing a response after a quiz was graded and returned, then reporting that there had been a mistake and requesting credit from the altered response[46]  Influencing the teacher to get more marks[43-45]  Getting technical help during practical exam[44]  Exchanging answers during an exam[40,43,44,46,47,49,52,54]  Moving labels or altering slides during an exam[54]  Passing an exam by using help from acquaintances[43,48,50]  Altering his or her grades in the official record[54]  Using crib notes[43,44,46-49,51,52,54]  Using mobile phone to exchange answers during an exam[43,45,48]  Arranging with administrative personnel to be assigned to a lenient examiner[48]  Paying a fellow student, or being paid by a fellow student for completion of coursework[43] |
|  | Lying | Unsatisfactory honesty/integrity[33]  Collusion[42]  Falsifies actions/information[25,37]  Giving false excuses when absent[40,43,44,46]  Lying about having ordered tests[41,53]  Giving false identification when challenged[26] |
|  | Data fabrication | Data fabrication[40]  Fabricating the whole or part of a patient’s history[40,41,45]  Altering or manipulating data (e.g., adjusting the data to obtain a significant result)[43,51]  Reporting a lab test or X-ray as “normal” during rounds when in actual ordered or knew it had not been[46,52]  Writing fake examination findings without performing it[41,44-46,49,52,55]  using auto-inserted data for vital signs[58]  using auto-inserted data for lab results[58]  using auto-inserted data for the medication list[58]  using templates for the entire note[58]  using templates for the physical or mental status exam[58]  using auto-inserted data for the problem list[58] |
|  | Data falsification | Data falsification[31,32,40,52]  Forging prescriptions[32]  Recording tasks that were not performed[53]  Falsifying references or a biography[52,55]  Falsifying lab data[46,53,55]  Writing clinical exam “normal" when you didn’t perform[40,51,55]  Documenting while signed in under an attending’s name[58]  Documenting while signed in under a resident’s name[58]  Forging signatures[26,40,42-45,48,49]  Using other people’s medical stamps[40]  Intentionally falsifying the test results or treatment records in order to disguise mistakes[43]  Falsifying references or grades on curriculum vitae[43]  Altering grades in official record[43]  Presenting work with the name of someone who did not participate in it[41] |
|  | Misrepresentation | Misrepresentation[25,35,57,59]  Being introduced as “doctor” to patients[29]  Not correcting someone who mistakes you for a physician[30] |
|  | Acting without patients’ consent | No consent for clinical examination of a patient[56,57] |
|  | Plagiarism | Copying text without appropriate attribution[26,40,42,43,47,60]  Copying elements of my own previous notes[43,58]  Turning in work done by someone else[43,46-49,51-54]  Allowing others to copy your work[35,51]  Copying and pasting elements of another provider’s notes in the electronic health record documentation (EHRD)[58]  Failing to correctly acknowledge a source (e.g., copying the text directly but only including the source in reference list)[43]  Citing sources that have not in fact been read in full[43] |
|  | Not obeying rules and regulations | Failing to obey rules & regulations[26]  Removing an assigned reference from the reserved shelf in the library, thereby preventing other students from gaining access to the information[43,46,52]  Acceptance of gifts[35,49]  Buying or selling hospital shifts[40,41]  Taking food that is not meant for students[29,30]  Eating or drinking in patient corridors[29]  Failing to follow proper infection control procedures[43,57]  Use of phones in restricted areas[61]  Asking someone to include you in the assistance list[41]  Inebriation at school events[26,30]  Arrest or criminal offence[26]  Significant misconduct[42]  Stealing or breaking things[62]  Committing a felony[32] |
| Disrespectful behaviour | Poor verbal/ nonverbal communication | Unsatisfactory respect[26,31,33]  Poor verbal communication[25,28,32]  Poor nonverbal communication[26,28]  Disrespectful communication by email[32]  Verbally expressed hostility, e.g. posing provocative questions in a challenging manner[66]  Fails to establish rapport[31]  Inadequate rapport with patients/families[25,37,56]  Speaking too casually in examination[28]  Threatening or verbally abusing a university employee or fellow student[43]  Showing outright hostility, malice or rudeness[39]  Hostile[27]  Rude[27]  Arrogant[37]  Manipulative, aggressive, and badgering of faculty[36]  Doesn’t respond to written requests to discuss low grades[36]  Failing to listen to patients’ opinion[26]  Rude or aggressive to fellow students or to staff, with confrontational, intimidating or arrogant behaviour[26]  Ignoring emails or other contacts from teaching or administrative staff[26]  Rudeness to colleague in presence of simulated patient[26]  Compromising ethical principles[35]  Belligerence[66] |
|  | Inappropriate clothing | Poor condition of white coats[29,30]  Untidy dress[29]  Wear white coats/scrubs out of the hospital[29]  Failure to maintain professional appearance and attire[25,28,30,37,42] |
|  | Disruptive behaviour in teaching sessions and exams | Negative responses in a sex education seminar[66]  Whispering animatedly about material that was obviously not of general educational value[66]  Negative responses in a sex education seminar[66]  Inappropriate behaviour in lecture[36]  Unnecessary interruption in class[34]  Dismissive or arrogant behaviour to other individuals during teaching[26]  Using offensive language during teaching sessions[26]  Failure to show respect for the examination process[28]  Writing rude/inappropriate comments on exam script[26] |
|  | Privacy and confidentiality violations | Fails to respect patient confidentiality[25,35,56]  Discussing patients in public spaces, including Facebook[29,30,63-65] |
|  | Inappropriate use of internet | Inappropriate use of social media[32]  Use Facebook or Google to research patients[67]  Discussing a clinical site in a negative light[64]  Discussing university in a negative light[64]  Discussing another health care worker in a negative light[64]  On line posting sexual-relational content, i.e. posting sexually suggestive/ explicit content or posting sexually provocative photographs of students, requesting inappropriate friendships with patients on Facebook, sexually suggestive comments[63,65]  On line posting negative content related to experiences in medical school, i.e. using profanity or other disparaging or discriminatory language in reference to specific faculty, courses or rotations, classmates, or medical school[63]  On line posting content like comments, photos and videos suggesting intoxication or illicit substance use[63,65] |
|  | Bullying | Verbal abuse[68,69]  Written abuse[68]  Physical abuse[43,68,69]  Behavioural abuse[68,69]  Subgroup formation[66]  Ignoring and excluding a peer student[62,68]  Deliberately damaging another students’ work[43]  Threatening others[62]  Spreading rumours[62]  Profanity[62]  Insulting[62] |
|  | Discrimination | Cultural and religious insensitivity[35]  Bias[35]  Discrimination[33] |
|  | Sexual harassment | Sexual harassment[35,43] |
| Poor self-awareness | Avoiding feedback | Unclear expectations or insufficient feedback by faculty or residents[30] |
|  | Lacking insight in own behaviour | Poor insight[28]  Lack of self-awareness[32,33]  Denying own performance[28]  Student failing to appreciate the effects of poor health on performance and seek support[26]  Work or attendance affected by health disorders such as depression[26]  Seems to feel put upon when asked to do authority[36]  Lack of insight into behaviour[26]  ‘Con artist ‘ (manipulative behaviour)[27] |
|  | Blaming external factors rather than own inadequacies | Blaming external factors rather than skill deficiencies for bad exam results[28]  Challenges everything[27]  Argumentative[31,37] |
|  | Not accepting feedback | Fails to accept responsibility for actions[25]  Resistant to accepting feedback[25,32,34,37,70]  Inability to incorporate feedback[31] |
|  | Resisting change | Diminished capacity for self-improvement[32,38,70]  Resistant to change[37]  Lack of effort towards self-improvement[37] |
|  | Not aware of limitations | Lack of awareness of one’s limitations[25,32,37]  Placing own learning above patient safety[57]  Acting beyond level of competence[30,43,56,57]  Discuss with patients information beyond your level of knowledge[29,30]  Inappropriate advice to a patient[26]  Giving other students inappropriate advice about clinical care[26]  Not respecting professional boundaries (deciding to visit a patient at home)[26]  Arrogant and overconfident[27,31,35,38]  Arrogant or abusive during stress[25]  Abuses student privileges[25]  Endorsed more than one unprofessional behaviours[49] |
|  | Not sensitive to another person’s needs | Lack of empathy[25-28,31,33,37]  Fail to elicit the patient’s perspective[26,28,30]  Abrupt and non-empathetic manner with patients[26]  Making derogatory comments about patients[30,39,56,57]  Putting own learning needs ahead of patient care, and thereby causing the patient discomfort[26,56,57]  Treat simulation patients as symptoms and diagnoses rather than as people with feelings and concerns[28]  Making fun of patients, peers, or physicians[26,29,30]  Inappropriate comments made to a patient in front of others[26]  Reporting an impaired colleague to faculty before approaching the individual[29]  Displays inappropriate interpersonal skills[27,31] |
