# Supplementary material for: Descriptors for unprofessional behaviours of medical students: a systematic review and categorisation
Source: BMC Med Educ. 2017 Sep 15;17:164. doi: 10.1186/s12909-017-0997-x (PMC5603020; doi:10.1186/s12909-017-0997-x)
Supplement: Supplementary file 5 — Findings mapped to GMC’s domains of concern. (DOCX 30 kb) [file 12909_2017_997_MOESM5_ESM.docx]

| **GMC Domain of concern** | **GMC Examples of behaviours**  (normative, not based on empirical research) | **Findings and categorisation**  (based on empirical research) |
| --- | --- | --- |
| **Persistent inappropriate attitude or behaviour** | Uncommitted to work or a lack of engagement with training, programme of study or clinical placements | Failure to engage/poor initiative/   - *General lack of commitment to teaching & learning activities and/or tutor meetings*[26]   Failure to engage/cutting corners   - *Poor reliability and responsibility* [25,31,33,34,37,38,42,70] - *Inadequate personal commitment to patients*[25] - *Accepts/seeks minimally acceptable level of performance*[25] - *Reluctance in pursuing clinically appropriate diagnostic and therapeutic steps, including avoiding admission, pressing for premature discharge, or otherwise cutting corners*[39] - *Lack of conscientiousness*[35] - *Avoids work*[27,32] - *Leaving the hospital during a shift*[41] |
|  | Neglect of administrative tasks | Failure to engage/Not meeting deadlines/   - *Failure to follow the timetable and/or get assignments signed off*[26,32] |
|  | Poor time management | Failure to engage/Not meeting deadlines/   - *Failure to follow the timetable and/or get assignments signed off*[26,32] |
|  | Non-attendance | Failure to engage/absent or late for assigned activities/   - *Lack of timeliness*[28-30,37] - *Unexplained/unauthorised absence*[26,27,29-32,42,43] |
|  | Poor communication skills | Disrespectful behaviour /Poor verbal or nonverbal communication   - *Poor verbal communication*[25,28,32] - *Poor nonverbal communication*[26,28] |
|  | Failure to accept and follow educational advice and unwillingness to learn from feedback given by others | Poor self-awareness/Not accepting feedback/   - *Resistant to accepting feedback*[25,32,34,37,70] - *Inability to incorporate feedback*[31] |
|  | Being rude to patients, colleagues or others | Disrespectful behaviour/Poor verbal or nonverbal communication   - *Rude*[27] |
|  | Unwillingness to learn from constructive feedback given by others | Poor self-awareness/Not accepting feedback/   - *Resistant to accepting feedback*[25,32,34,37,70] - *Inability to incorporate feedback*[31] |
|  | Being disruptive in teaching sessions or the training environment | Disrespectful behaviour/Disruptive behaviour in teaching sessions and exams/   - *Inappropriate behaviour in lecture*[36] - *Unnecessary interruption in class*[34] - *Dismissive or arrogant behaviour to other individuals during teaching*[26] - *Using offensive language during teaching sessions*[26] |
|  | Challenging behaviour towards clinical teachers or not accepting criticism | Poor self-awareness/Blaming external factors rather than own inadequacies   - *Blaming external factors rather than skill deficiencies for bad exam results*[28] - *Challenges everything*[27] - *Argumentative*[31,37] |
|  | Failing to answer or respond to communications | Disrespectful behaviour /Poor verbal ornonverbal communication   - *Ignoring emails or other contacts from teaching or administrative staff*[26] |
| **Failing to demonstrate good medical practice** | Misuse of social media, such as criticising placement providers | Disrespectful behaviour/Inappropriate use of social media   - *Discussing university in a negative light*[64] |
|  | Breach of confidentiality | Disrespectful behaviour/Privacy and confidentiality violations/   - *Fails to respect patient confidentiality*[25,35,56] - *Discussing patients in public spaces, including Facebook*[29,30,63-65] |
|  | Misleading patients about their care or treatment | Disrespectful behaviour /Poor verbal/ nonverbal communication   - *Inadequate rapport with patients/families*[25,37,56] |
|  | Culpable involvement in a failure to obtain proper consent from a patient | Dishonest behaviour /Acting without required consent   - *No consent for clinical examination of a patient*[56,57] |
|  | Sexual, racial or other forms of harassment or bullying | Disrespectful behaviour /Bullying   - *Verbal abuse*[68,69] - *Written abuse*[68] - *Physical abuse*[43,68,69] - *Behavioural abuse*[68,69] - *Subgroup formation*[66] - *Ignoring and excluding a peer student*[62,68] - *Deliberately damaging another students’ work*[43] - *Threatening others*[62] - Spreading rumours[62] - Profanity[62] - *Insulting*[62]   Disrespectful behaviour /sexual harassment   - *Sexual harassment* [35,43] |
|  | Inappropriate examinations or failure to keep appropriate boundaries in behaviour | Disrespectful behaviour/Disruptive behaviour in teaching sessions and exams   - *Failure to show respect for the examination process[28]* - *Writing rude/inappropriate comments on exam script[26]* |
|  | Unlawful discrimination | Disrespectful behaviour /Discrimination   - *Discrimination*[33] |
| **Drug or alcohol misuse** | Driving under the influence of alcohol or drugs | Not found |
|  | Abusing prescription medication |  |
|  | Alcohol consumption that affects clinical work, the work environment, or performance in the educational environment |  |
|  | Dealing, possessing, supplying or misusing drugs, even if there are no legal proceedings – this may include legal highs |  |
|  | A pattern of excessive misuse of alcohol |  |
| **Cheating or plagiarising** | Cheating in examinations | Dishonest behaviour/Cheating in exams   - *Cheating in exams[32,40,45,50,53,55]* - *Gaining illegal access to examination questions[40,43-48,51,53,54]* - *Paying someone to change a grade[41,45,48]* - *Let someone else sit for your exams or taking a test or a part of a test for someone else[46,47,51,52,54]* - *Observing a student copying from another student during an examination and doing nothing with the information[46]* - *Changing a response after a quiz was graded and returned, then reporting that there had been a mistake and requesting credit from the altered response[46]* - *Influencing the teacher to get more marks[43-45]* - *Getting technical help during practical exam[44]* - *Exchanging answers during an exam[40,43,44,46,47,49,52,54]* - *Moving labels or altering slides during an exam[54]* - *Passing an exam by using help from acquaintances[43,48,50]* - *Altering his or her grades in the official record[54]* - *Using crib notes[43,44,46-49,51,52,54]* - *Using mobile phone to exchange answers during an exam[43,45,48]* - *Arranging with administrative personnel to be assigned to a lenient examiner[48]* - *Paying a fellow student, or being paid by a fellow student for completion of coursework[43]* |
|  | Signing peers into taught sessions from which they are absent | Dishonest behaviours/ Lying   - *Falsifies actions/information*[25,37] - *Unsatisfactory honesty/integrity*[33]   Dishonest behaviours/Data falsification/   - *Data falsification*[31,32,40,52] - *Forging signatures*[26,40,42-45,48,49] |
|  | Passing off the work of others as your own | Dishonest behaviours/ Plagiarism   - *Turning in work done by someone else*[43,46-49,51-54] |
|  | Sharing with fellow students or others, details of questions or tasks from exams you have taken | Dishonest behaviour/Cheating in exams   - *Gaining illegal access to examination questions*[40,43-48,51,53,54] |
|  | Forging a supervisor’s name or falsifying feedback on assessments, logbooks or portfolios | Dishonest behaviour/Lying   - *Falsifies actions/information*[25,37]   Dishonest behaviour/data falsification   - *Forging signatures*[26,40,42-45,48,49] |
| **Dishonesty or fraud, including dishonesty outside the professional role** | Falsifying research | Dishonest behaviour/data falsification   - *Data falsification*[31,32,40,52] |
|  | Committing financial fraud | Not found |
|  | Creating fraudulent CVs or other documents | Dishonest behaviour/data falsification   - *Falsifying references or grades on curriculum vitae*[43] |
|  | Misrepresentation of qualifications | Dishonest behaviour/data falsification   - *Falsifying references or grades on curriculum vitae*[43]   Dishonest behaviour /Misrepresentation   - *Misrepresentation*[25,35,57,59] - *Not correcting someone who mistakes you for a physician*[30] |
|  | Falsifying signatures on documents such as portfolios | Dishonest behaviour/data falsification  • *Forging signatures*[26,40,42-45,48,49] |
|  | Failure to declare relevant misconduct or health issues to your medical school or university | Not found |
|  | Wilful withholding or misrepresentation of health issues (for example, blood-borne viruses) | Not found |
| **Aggressive, violent or threatening behaviour** | Assault | Disrespectful behaviour /Bullying  • *Physical abuse*[43,68,69]  • *Deliberately damaging another students’ work*[43] |
|  | Physical violence | Disrespectful behaviour /Bullying  • *Physical abuse*[43,68,69] |
|  | Bullying | Disrespectful behaviour /Bullying  • *Verbal abuse*[68,69]  • *Written abuse*[68]  • *Subgroup formation*[66]  • *Ignoring and excluding a peer student*[62,68]  • *Deliberately damaging another students’ work*[43]  • *Threatening others*[62]  • *Spreading rumours*[62]  • *Profanity*[62] |
|  | Harassment | Disrespectful behaviour /Bullying   - *Insulting*[62] |
|  | Stalking | Disrespectful behaviour /Bullying   - *Behavioural abuse*[68,69] |
|  | Online bullying or trolling | Disrespectful behaviour/Inappropriate use of social media   - *On line posting sexual-relational content, i.e. posting sexually suggestive/ explicit content or posting sexually provocative photographs of students, requesting inappropriate friendships with patients on Facebook, sexually suggestive comments*[63,65] |
| **Any caution or conviction** | Possessing, dealing or supplying illegal drugs | Dishonest behaviours/Not obeying rules and regulations/   - *Arrest or criminal offence*[26] - *Significant misconduct*[42] |
|  | Theft | Dishonest behaviours/Not obeying rules and regulations/   - *Stealing or breaking things*[62] |
|  | Physical violence | Dishonest behaviours/Not obeying rules and regulations/   - *Significant misconduct*[42]   Disrespectful behaviour /Bullying   - *Physical abuse*[43,68,69] |
|  | Fare avoidance | Dishonest behaviours/Not obeying rules and regulations/   - *Stealing or breaking things*[62] |
|  | Financial fraud | Dishonest behaviours/Not obeying rules and regulations/   - *Failing to obey rules & regulations*[26] - *Acceptance of gifts*[35,49] - *Buying or selling hospital shifts*[40,41] - *Significant misconduct*[42] |
|  | Child pornography | Dishonest behaviours/Not obeying rules and regulations/   - *Arrest or criminal offence*[26] - *Significant misconduct*[42] |
|  | Child abuse or any other abuse | Dishonest behaviours/Not obeying rules and regulations/   - *Significant misconduct*[42] |
|  | Sexual offences | Dishonest behaviours/Not obeying rules and regulations/   - *Significant misconduct*[42] |
| **Health concerns and insight or management of these concerns** | Failure to seek appropriate treatment or advice from an independent and appropriately qualified healthcare professional | Poor self-awareness/Not aware of limitations   - *Lack of awareness of one’s limitations*[25,32,37] |
|  | Failure to follow the requirement to tell your medical school or university if you have a serious health condition | Lacking insight in own behaviour/Poor insight[28]   - *Work or attendance affected by health disorders such as depression*[26] |
|  | Refusal to follow medical advice or care plans, or to comply with arrangements for monitoring and reviews | Not found |
|  | Failure to comply with reasonable adjustments to ensure patient safety | Poor self-awareness/Not aware of limitations   - *Placing own learning above patient safety*[57] |
|  | Failure to recognise limits and abilities or lack of insight into health concerns | Poor self-awareness/Not aware of limitations   - *Lack of awareness of one’s limitations*[25,32,37] |
|  | Failure to be immunised against common serious communicable diseases (unless contraindicated) | Dishonest behaviour/Not obeying rules and regulations   - *Failing to follow proper infection control procedures*[43,57] |

The following found descriptors could not be mapped to the GMC Domains of concern:

poor initiative

general disorganisation

poor teamwork

language difficulties

data-fabrication

inappropriate clothing

avoiding feedback

resisting change

not being sensitive to other person’s needs
